# Identification of Potential New Protein Vaccine Candidates through Pan-Surfomic Analysis of Pneumococcal Clinical Isolates from Adults

**DOI:** 10.1371/journal.pone.0070365

**Published:** 2013-07-23

**Authors:** Alfonso Olaya-Abril, Irene Jiménez-Munguía, Lidia Gómez-Gascón, Ignacio Obando, Manuel J. Rodríguez-Ortega

**Affiliations:** 1 Departamento de Bioquímica y Biología Molecular, Universidad de Córdoba; Campus de Excelencia Internacional CeiA3; Hospital Universitario Reina Sofía, Córdoba; Instituto Maimónides de Investigación Biomédica de Córdoba (IMIBIC), Córdoba; and Red Española de Investigación en Patología Infecciosa (REIPI), Instituto de Salud Carlos III, Madrid, Spain; 2 Departamento de Sanidad Animal, Universidad de Córdoba, Córdoba, Spain; 3 Sección de Enfermedades Infecciosas Pediátricas e Inmunopatología, Hospital Universitario Infantil Virgen del Rocío, Sevilla, Spain; Instituto Butantan, Brazil

## Abstract

Purified polysaccharide and conjugate vaccines are widely used for preventing infections in adults and in children against the Gram-positive bacterium *Streptococcus pneumoniae*, a pathogen responsible for high morbidity and mortality rates, especially in developing countries. However, these polysaccharide-based vaccines have some important limitations, such as being serotype-dependent, being subjected to losing efficacy because of serotype replacement and high manufacturing complexity and cost. It is expected that protein-based vaccines will overcome these issues by conferring a broad coverage independent of serotype and lowering production costs. In this study, we have applied the “shaving” proteomic approach, consisting of the LC/MS/MS analysis of peptides generated by protease treatment of live cells, to a collection of 16 pneumococcal clinical isolates from adults, representing the most prevalent strains circulating in Spain during the last years. The set of unique proteins identified in all the isolates, called “pan-surfome”, consisted of 254 proteins, which included most of the protective protein antigens reported so far. In search of new candidates with vaccine potential, we identified 32 that were present in at least 50% of the clinical isolates analyzed. We selected four of them (Spr0012, Spr0328, Spr0561 and SP670_2141), whose protection capacity has not yet been tested, for assaying immunogenicity in human sera. All of them induced the production of IgM antibodies in infected patients, thus indicating that they could enter the pipeline for vaccine studies. The pan-surfomic approach shows its utility in the discovery of new proteins that can elicit protection against infectious microorganisms.

## Introduction

*Streptococcus pneumoniae* is a Gram-positive bacterium that can be found as a commensal in the human respiratory tract and that, under appropriate conditions, is pathogenic, being able to cause high morbidity and mortality [1]. This microorganism is a leading cause of mucosal diseases such as otitis media, sinusitis and pneumonia and is a prominent pathogen in invasive diseases including bacteremia, meningitis, and sepsis [2]. Pneumococcal disease disproportionally affects young children and the elderly although it may occur in all age groups and with higher frequency among patients with co-morbid conditions. It has been estimated that approximately 800,000 children die each year due to pneumococcal disease and >90% of these deaths occur in developing countries [3]. Burden of disease due to pneumococcal diseases, generally related to pneumonia, is also high among adults in developed countries with around 25,000 deaths per year in the United States in adults over 50 years of age and significant mortality and long-term effects on quality of life in European countries [4], [5].

Prevention of pneumococcal disease by immunization has long been considered a major goal that could help to reduce the burden of pneumococcal diseases and to control antimicrobial resistance rates [6], [7]. Two types of pneumococcal vaccines are available in the market, both based on the capsule polysaccharide: pneumococcal purified polysaccharide vaccine and conjugate vaccines, in which polysaccharides are conjugated to a protein carrier capable of recruiting CD4^+^ T-cells, increasing immunogenicity in young children [8]. The first type is mainly used in adults, covering 23 capsule serotypes (Pneumovax 23V) that represent about 80% of the most prevalent pneumococcal disease-causing ones in children and adults in the USA [9]. A pneumococcal conjugate vaccine covering 7 serotypes (PCV7) was initially licensed for exclusive use in children, and new vaccines with broader serotype coverage (10V and 13V) were later developed. The 13-valent pneumococcal conjugate vaccine (PCV13) has been approved for prevention of invasive disease (FDA and EMEA) and pneumonia (FDA) caused by PCV13 serotypes among adults aged 50 years and older, and was recently recommended for adults aged ≥19 years with immunocompromising conditions in the United States by ACIP [10].

Although it seems that Pneumovax-23 protects effectively against invasive pneumococcal disease (IPD) in healthy adults, its efficacy in high-risk groups and against other outcomes (pneumonia, mortality) is less clear [1]. In addition, and together with conjugate vaccines, they present some important limitations [11]: i) coverage is serotype-dependent, not covering the majority of the 93 capsule serotypes described so far; ii) coverage is designed on the basis of the most prevalent serotypes identified in developed countries and may be less effective in developing countries; iii) vaccine effectiveness may decrease in the long term due to non-vaccine serotype replacement [12]; iv) high manufacturing complexity and cost make these vaccines less accessible to developing countries; and v) genomic factors other than capsular determinants may modulate virulence, and therefore it has been suggested that a vaccine based on genetic factors other than serotype may be necessary especially for otitis media and nonbacteremic pneumonia [13]. Protein-based vaccines theoretically offer advantages over those based on the capsule polysaccharides, by allowing them to overcome the previously cited problems: targeting conserved antigens in a serotype-independent way, covering a broader pneumococcal biotype population, and lowering cost of production [14]. Here, surface proteins are ideal as they have the highest chance of raising an effective immune response. So far, numerous pneumococcal proteins have shown protection against infection in animal models, but most of them are still in clinical trials. Proteomics provides excellent platforms and strategies to identify in a fast and reliable way the set of proteins expressed on the surface of pathogenic microorganisms. To this regard, the “shaving” approach –consisting of treating live cells with proteases, followed by LC/MS/MS analysis of the generated peptides– has become a highly valuable tool when searching for protein vaccine candidates [15], [16]. In this study, we have screened a collection of pneumococcal clinical isolates from adults by defining its “pan-surfome”, (i.e. the whole set of expressed surface proteins), in order to identify which proteins that have not been tested so far in animal models for protection against infection could enter the vaccine pipeline in future studies.

## Materials and Methods

### Ethics Statement for Human Sera Sampling and Use

This research was performed according to the principles expressed in the Declaration of Helsinki. All human sera were obtained from patients >8 years old admitted to Hospital Universitario Infantil Virgen del Rocío (HUIVR) in Seville, Spain. Sera were drawn either from patients with a diagnosis of pneumococcal infection, determined by isolation of the microorganism from a sterile site (blood or pleural fluid) according to standardised protocols or from healthy control children aged >8 years old. All sera from patients were obtained within ten days of hospital admission. Written informed consent was obtained from parents or legal guardians of participating children and the Hospital Universitario Virgen del Rocío Ethic Committee approved the study (code no. 010470, certificate no. 14/2010), for sera to be used within the project in which this work was designed.

### Bacterial Strains and Growth

The 16 *S. pneumoniae* strains used in this study isolated from adult patients corresponded to 9 different capsule serotypes **(** **Table 1** **)**. All the strains were maintained at −80°C, plated on Columbia blood agar base containing 6% (v/v) sheep blood and grown in a chemically-defined medium (CDM) [17] supplemented with 20 μg/ml ethanolamine (CDM+EA) as source of aminoalcohol, at 37°C and 5% CO_2_ until OD_600_ of 0.25 (mid-exponential phase) was reached.

**Table 1:** Sequence types (STs) and serotypes among 16 *Streptococcus pneumoniae* invasive isolates recovered from adult patients.

Isolate	ST	Allelic profile (MLST)*	Serotype	PMEN clone**
1	2480	12,8,4,5,18,58,18	9V	
2	191	8,9,2,1,6,1,17	7F	Netherlands^7F^-39
3	191	8,9,2,1,6,1,17	7F	Netherlands^7F^-39
4	162	7,11,10,1,6,8,14	14	SLV-Spain^9V^-3***
5	228	12,8,1,5,17,4,20	1	
6	433	1,1,4,1,18,58,17	19A	
7	306	12,8,13,5,16,4,20	1	Sweden^1^-28
8	289	16,12,9,1,41,33,33	5	Colombia^5^-19
9	7340	2,5,36,12,17,21,271	8	
10	1201	1,5,1,12,17,3,8	19A	
11	156	7,11,10,1,6,8,1	14	Spain^9V^-3
12	180	7,15,2,10,6,1,22	3	Netherlands^3^-31
13	53	2,5,1,11, 16,3,14	8	
14	557	7,11,10,1, 6,58,1	9V	SLV-Spain^9V^-3***
15	1223	16,12,9,1,6,33,33	5	SLV-Colombia^5^-19***
16	989	12,5,89,8,6,112,14	12F	

* Allelic profiles for each gene in multilocus sequence typing (MLST) are presented in the following order: *aroE, gdh, gki, recP,spi, xpt and ddl.*

** PMEN clones are global clones recognized by pneumococcal molecular epidemiology network.

*** SLV = single locus variant (i.e. differs at only one MLST locus and thus is a closely-related genotype).

### Molecular Genotyping

MLST was performed using standard methodology [18]. In brief, internal fragments of 7 housekeeping genes (*aroE, gdh, gki, recP, spi, xpt* and *ddl*) were amplified by polymerase chain reaction and sequenced on each strand. Conventional primers were used, whose sequences are available at the MLST database (http://www.mlst.net). Alleles were assigned by comparing the sequence at each locus to all known alleles at that locus, and the combination of 7 alleles determined the sequence type (ST). Allele and ST designations were made using the MLST website, hosted at Imperial College London, and funded by the Wellcome Trust.

### “Shaving” of Live Pneumococcal Cells with Trypsin

Generation and recovery of tryptic peptides from “shaved” cells was carried out as described in [19] without modifications. Briefly, 100 ml of cultures were centrifuged at 3,500×*g* for 10 min at 4°C, and the pelleted bacteria washed twice with PBS. Cells were resuspended in one-hundredth volume of PBS/30% sucrose (pH 7.4). Tryptic digestions were performed with 5 μg trypsin (Promega) for 30 min at 37°C. The digestion mixtures were centrifuged at 3,500×*g* for 10 min at 4°C, and the supernatants (the “surfomes” containing the peptides) were filtered using 0.22-μm pore-size filters (Milipore). Surfomes were re-digested with 2 μg trypsin overnight at 37°C with top-down agitation. Salts were removed prior to analysis, using Oasis HLB extraction cartridges (Waters). Peptides were eluted with increasing concentrations of acetonitrile/0.1% formic acid, according to manufacturer’s instructions. Peptide fractions were concentrated with a vacuum concentrator (Eppendorf), and kept at −20°C until further analysis.

### LC/MS/MS Analysis

All analyses were performed with a Surveyor HPLC System in tandem with an LTQ-Orbitrap mass spectrometer (Thermo Fisher Scientific, San Jose, USA) equipped with nanoelectrospray ionization interface (nESI), as described [19]. The separation column was 150 mm×0.150 mm ProteoPep2 C18 (New Objective, USA) at a postsplit flow rate of 1 µl/min. For trapping of the digest a 5 mm×0.3 mm precolumn Zorbax 300 SB-C18 (Agilent Technologies, Germany) was used. One fourth of the total sample volume, i.e. 5 µl, was trapped at a flow rate of 10 µl/min for 10 minutes and 5% acetonitrile/0.1% formic acid. After that, the trapping column was switched on-line with the separation column and the gradient was started. Peptides were eluted with a 60-min gradient of 5–40% of acetonitrile/0.1% formic acid solution at a 250 nl/min flow rate. All separations were performed using a gradient of 5–40% solvent B for 60 minutes. MS data (Full Scan) were acquired in the positive ion mode over the 400–1,500 m/z range. MS/MS data were acquired in dependent scan mode, selecting automatically the five most intense ions for fragmentation, with dynamic exclusion set to on. In all cases, a nESI spray voltage of 1.9 kV was used.

### Protein Identification by Database Searching

Tandem mass spectra were extracted using Thermo ProteomeDiscoverer 1.0 (Thermo Fisher Scientific). Charge state deconvolution and deisotoping were not performed. All MS/MS samples were analyzed using Sequest (Thermo Fisher Scientific, version v.27), applying the following search parameters: peptide tolerance, 10 ppm; tolerance for fragment ions, 0.8 Da; b- and y-ion series; oxidation of methionine and deamidation of asparagine and glutamine were considered as variable modifications; maximum trypsin missed cleavage sites, 3. The raw data were searched against an in-house joint database containing the protein sequences from all the sequenced and annotated *S. pneumoniae* strains available at the NCBI ftp site. Peptide identifications were accepted if they exceeded the filter parameter Xcorr score *vs* charge state with SequestNode Probability Score (+1 = 1.5, +2 = 2.0, +3 = 2.25, +4 = 2.5). With these search and filter parameters, no false-positive hits were obtained. For proteins identified from only one peptide, fragmentations were checked manually. Strain R6 was used as reference for providing the accession numbers of the identified proteins; whenever a protein belonging to another strain was found, homology with a corresponding protein of strain R6 was given by using proteinBLAST. If homology with R6 or TIGR4 proteins was not observed, then the protein accession numbers of the other strains were used.

### Bioinformatic Prediction of Protein Subcellular Localization

Primary predictions of subcellular localization were assigned by using the web-based algorithm LocateP (http://www.cmbi.ru.nl/locatep-db/cgi-bin/locatepdb.py). They were contrasted by several feature-based algorithms: TMHMM 2.0 (http://www.cbs.dtu.dk/services/TMHMM-2.0) for searching transmembrane helices; SignalP 3.0 (http://www.cbs.dtu.dk/services/SignalP) for type-I signal peptides: those proteins containing only a cleavable type-I signal peptide as featured sequence were classed as secreted; LipoP (http://www.cbs.dtu.dk/services/LipoP) for identifying type-II signal peptides, which are characteristic of lipoproteins. Topological representations of membrane proteins were performed with the web-based TOPO2 software (http://www.sacs.ucsf.edu/TOPO2/). GO annotations were retrieved from the UniProt Knowledgebase (http://www.uniprot.org/).

### Western Blot Analysis

Immunoreactivity of pneumococcal proteins was performed by Western blotting. 2 μg of pneumococcal recombinant proteins Spr0328 and Spr0561, 5 μg of pneumococcal recombinant proteins Spr0012, Spr0121, and SP670_2141 (produced as GST-tag recombinant fragments using the pSpark® vector, Canvax Biotech, Córdoba, Spain, and expressed in *E. coli* BL21. See Dataset S1 for further details), 1 μg trypsin (Promega) as negative control, and 1 μg of total protein extract of pneumococcus as positive control were separated by 12% SDS-PAGE gels and transferred to nitrocellulose membranes (Life Sciences). Non-specific sites were blocked by incubation with 5% non-fat milk in T-TBS for 1 h. After two washes with T-TBS, a second 1-h incubation of the membranes with patient sera, diluted 1∶100 in T-TBS for IgG detection and 1∶1,000 for IgM detection, was carried out. Interference of IgG antibodies in IgM detection was avoided using GullSORB ™ (Meridian Bioscience, Inc.), according to manufacturers’ instructions. Secondary antibodies consisted of rabbit anti-human IgG or rabbit anti-human IgM conjugated to horseradish peroxidase (Sigma), diluted 1∶5,000 or 1∶2,500 in TBS, respectively, for 1 h. Then, the membranes were washed three times with TBS and developed with ECL Plus Western Blotting Dectection System (GE Healthcare) according to the manufacturer’s instructions.

## Results and Discussion

We analyzed 16 clinical isolates collected from adult patients with invasive pneumococcal disease (IPD). Nine different serotypes were identified. These serotypes included the five most prevalent ones found in IPD among older people in Spain during the most recent years (19A, 3, 7F, 14 and 1), serotypes 9V and 19F that had been circulating significantly in this population in pre- and post-PCV7 periods, as well as serotypes 8 and 12F that were generally restricted to adults and with epidemic potential [20], [21]. We genotyped all clinical isolates by MLST, because genetic diversity of pneumococcal surface proteins depends on non-capsular genomic background. Fifteen clonal types were found including several major global clones recognized by the Pneumococcal Molecular Epidemiology Network (PMEN) (http://www.sph.emory.edu/PMEN) and genetically related genotypes as shown in Table 1. The most relevant among them were: ST156 (Spain^9V^-3), one of the most successful clones worldwide that has been especially responsible for the expansion of serotype 14 in IPD in various countries during recent years [22]; ST306 (Sweden^1^-28), a highly circulating genotype associated with the increase in the incidence of pleural empyema in children and adults in the last decade [23], [24]; ST289 (Colombia^5^-19), a highly relevant genotype identified in IPD both in developing and developed countries and associated with major local geographical outbreaks [25], [26]; ST180 (Netherlands^3^-31), most commonly found clonal type in serotype 3 that is associated to increased risk of mortality in pneumococcal pneumonia [27], [28]; and finally, ST191 (Netherlands^7F^-39), the dominant clonal type associated to the emerging serotype 7F during the post-PCV7 period [29].

Interaction between cells and their environment is critically mediated by surface proteins. In the case of pathogenic bacteria, these molecules are often virulence factors or responsible for pathogenesis. Since they also interact with the immune system, many of them are highly immunogenic and are thus ideal targets for novel vaccine discovery and development [30]. Protein-based vaccines composed of a unique antigen or a combination of them in a single formulation, may overcome the challenges remaining with polysaccharide-based vaccines, such as serotype replacement and high cost, thus making prophylaxis more affordable for resource-limited populations [11].

To this regard, proteomics has been revealed as the best means for high-throughput screening of large amounts of proteins for any biological purpose, and in particular for defining the set of surface-expressed proteins on a given organism. Several proteomic analyses have been carried out on the pneumococcus, targeting either the membrane fraction [31]–[33] or the cell wall-attached proteins [34], [35], but these biochemical fractionation-based methods have several limitations: they are relatively slow, membrane proteins are not always well resolved in polyacrylamide gels and topology information is lost [19], [36]. The “shaving” approach has become a powerful way to identify the set of surface proteins expressed on a given organism (the “surfome”), most of which are normally highly immunogenic as shown by different immunochemical techniques [15], [16], [37], and provides new candidates that elicit protective activity against infection [38]. We have previously set up this strategy in the pneumococcus [19], showing that the procedure enables to define the “pan-surfome” of a collection of clinical isolates, in order to select common proteins to all or most strains [37], [39].

**Figure 1:**
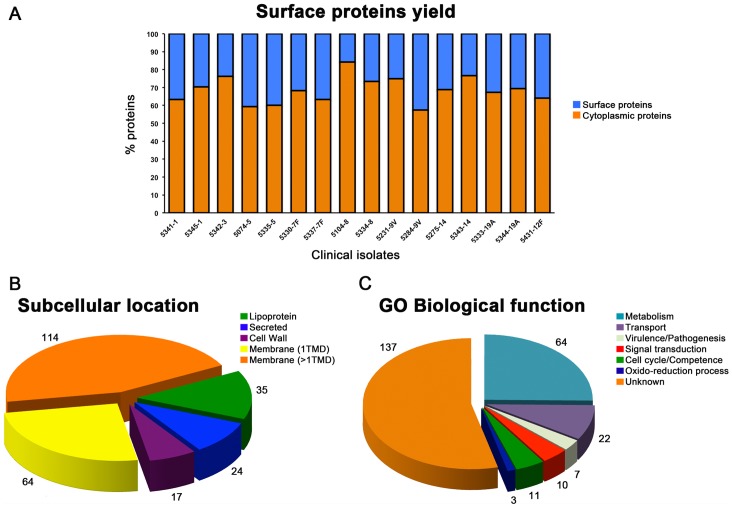
Pan-surfomic analysis of pneumococcal adult clinical isolates. A) Yield of surface protein identification after trypsin treatment in each of the 16 analyzed isolates. B) Subcellular localization of the predicted surface proteins, according to LocateP (TMD: transmembrane domain). C) Gene Ontology annotations of the biological functions of the identified surface proteins. Numbers in panels B and C represent the proteins belonging to each category.

We applied the optimized “shaving” protocol for pneumococcus to the collection of adult clinical isolates, growing cells in a chemically-defined medium with ethanolamine (CDM+EA) and digesting them with trypsin for 30 min at 37°C. As already described by our group [19], we have compared the efficiency of the “shaving” procedure in different culture media. In the complex Todd-Hewitt broth, 44 surface proteins were identified at the described trypsin digestion conditions, compared to 32 found in CDM+EA; however, a higher percentage of cytoplasmic proteins was found in THB than in CDM+EA (83% vs 61%). Moreover, these differences were even higher when considering identified peptides (in THB, only 15% corresponded to surface proteins, whereas in CDM+EA this percentage was 43%). A total of 254 surface proteins were identified in the set of the 16 isolates (Table S1 and Datasets S2 and S3), and the yields of surface protein identification ranged between 20% and 40%, approximately (**Figure 1A**). Within the different categories of surface proteins recognized for Gram-positive bacteria, and particularly for streptococci, most of them (114+64 = 178, i.e. 70%) had some transmembrane domain, 24 (9.4%) were predicted by LocateP as secreted, 35 (13.8%) as lipoproteins and 17 (6.7%) as possessing an LPXTG-anchoring motif to the cell wall (**Figure 1B**). **Figure 1C** shows the GO annotations according to their biological function, with a high number of proteins with unpredicted functions, which make them highly interesting for further studies on molecular characterization, participation in virulence or pathogenesis, or assay of protection activity.

The “shaving” approach has already demonstrated its power for identifying most of the previously discussed protective protein antigens in other streptococcal species, even when the number of clinical isolates used is low [15], [16]. Our pan-surfomic analysis included 40 out of the 44 proteins that have shown to induce protection against pneumococcal infection [34], [40]–[64] (**Table 2**). Among these proteins, 13 are predicted as cytoplasmic. Although many proteins pertaining to this category have been reported both to be surface-located and to induce protection, the targets of our analysis were those ones for which subcellular localization algorithms predicts undoubtedly to be exported outside the cell. Interestingly, we identified in a high proportion of clinical isolates some of the best candidates described so far: the membrane protein PspA [53] and the predicted extracellular proteins CbpA [41] and PcsB [54]. Three out of the four protective antigens not detected in our study were the pilin proteins RrgA, RrgB and RrgC. Previous works have shown that Gram-positive pilus subunit proteins are resistant to trypsin treatment [65], and proteolysis with a non-specific protease such as proteinase K is required to identify them [15], [16], [66], [67].

**Table 2:** Identification of protective protein antigens against *Streptococcus pneumoniae* infection already described in literature.

Protein family	Antigen	Locus	Location	References	Found in # strains
Choline-binding proteins	PspA	spr0121	1TMD	[53]	14/16
	CbpA (PspC)	spr1995	Secreted	[41]	16/16
	PcpA	spr1945	Secreted	[45]	5/16
	LytA	spr1754	Cytoplasmic	[47]	3/16
	LytB	spr0867	>1TMD	[52]	7/16
Toxins	Ply	spr1739	Cytoplasmic	[40]	6/16
Sortase and sortase-dependent proteins	SrtA	spr1098	1TMD	[44]	4/16
	RrgA	SP_0462	Cell Wall	[43]	0/16
	RrgB	SP_0463	Cell Wall	[43]	0/16
	RrgC	SP_0464	Cell Wall	[43]	0/16
	NanA	spr1536	Cell Wall	[48]	13/16
	SphtrA	spr2045	1TMD	[52]	11/16
ABC transporter proteins	PiaA (ABC-SBP)	spr0934	Lipoprotein	[46]	2/16
	PiuA (fecE)	spr1686	Cytoplasmic	[46]	2/16
	PsaA	spr1494	>1TMD	[51]	2/16
	PotD	spr1243	1TMD	[50]	1/16
	sp0148 (ABC-SBP)	spr0146	Lipoprotein	[49]	5/16
Enzymatic proteins	ClpP	spr0656	Cytoplasmic	[42]	1/16
	StkP	spr1577	1TMD	[54]	1/16
	FBA	spr0530	Cytoplasmic	[34]	13/16
	gapA	spr1825	Cytoplasmic	[34]	16/16
	eno	spr1036	Cytoplasmic	[56]	16/16
	tig	spr0362	Cytoplasmic	[56]	9/16
	ldh	spr1100	Cytoplasmic	[56]	11/16
	pykF	spr0797	Cytoplasmic	[56]	15/16
	pgk	spr0441	Cytoplasmic	[56]	15/16
	gnd	spr0335	Cytoplasmic	[34]	13/16
Histidine triad proteins	PhtA	spr1061	Lipoprotein	[55]	6/16
	PhtB	spr1060	Lipoprotein	[55]	9/16
	PhtD	spr0907	1TMD	[55]	14/16
	PhtE	spr0908	Secreted	[55]	10/16
Others	PcsB	spr2021	Secreted	[54]	12/16
	PppA	spr1430	Cytoplasmic	[59]	6/16
	PpmA (PrsA)	spr0884	Lipoprotein	[62]	8/16
	SlrA	spr0679	Cytoplasmic	[60]	11/16
	AmiA	spr1707	Lipoprotein	[61]	5/16
	AliB	spr1382	Lipoprotein	[61]	9/16
	AliA	spr0327	Lipoprotein	[61]	7/16
	PfbB	spr0075	Cell Wall	[63]	8/16
	PsrP	sp_1772	Cell Wall	[64]	0/16
	spr0785	spr0785	Cytoplasmic	[49]	1/16
	spr1176	spr1176	Cytoplasmic	[58]	1/16
	spr2010	spr2010	>1TMD	[58]	1/16
	spr1875	spr1875	1TMD	[57]	8/16

To be considered as a promising vaccine candidate, a given protein should ideally be surface-exposed, highly expressed, and distributed as widely as possible [36] in order to overcome the limitations of serotype-dependent polysaccharide vaccines [14]. However, experimentally identifying a high number of proteins common to all the analyzed strains, can be a hard task. Actually, Dreisbach et al. found in *Staphylococcus aureus* that only 7 proteins (less than 10% of total identified) were common to the 4 isolates analyzed [39]. We have reported very similar results in a low number of pneumococcal strains (12 proteins, i.e. 10.5% of total, in 5 isolates). But, as the number of analyzed isolates increases, the probability of finding some proteins common to all of them diminishes. In fact, we did not find any common protein to the 39 *Streptococcus suis* clinical isolates in a recent report [37]. In the search for pneumococcal surface proteins to be proposed as potential new vaccine candidates, we established a threshold for the presence of a protein in at least 50% of the analyzed clinical isolates; otherwise, a very stringent criterion would lead us to identify the already described protective antigens. Thus, 32 proteins surpassed this threshold (**Table 3**). As expected, cell wall-anchored, for which 10 proteins were identified, was the category of surface proteins with the highest number of items above this limit. Spr0075 was just identified in 50% of the isolates; the other 9 cell-wall proteins were present in ≥11 strains. Three lipoproteins were also found according to the selected criterion, including the protective antigens PhtD and AliB (see **Table 3**), as well as 7 proteins with one transmembrane domain (that included protective antigens PspA and Spr1875) and other 7 proteins with more than one transmembrane helix. Finally, 5 predicted proteins to be secreted to the extracellular milieu were also found, including the highly protective candidates CbpA and PcsB (annotated in the R6 genome as Spr2021). As cell-wall proteins with the LPXTG-anchoring motif are those most protruding and exposed on the streptococcal surface [68], they are expected to be found most frequently within a collection of clinical isolates. Actually, we have reported very similar results in the pan-surfome of 39 *S. suis* clinical isolates, where 13 cell wall proteins (out of 17 identified, and out of 20 predicted in the database used) were identified in ≥50% of strains [37].

**Table 3:** Surface proteins identified in ≥50% clinical isolates by “shaving” *Streptococcus pneumoniae* cells followed by LC/MS/MS analysis.

		Clinical Isolates																
Locus	Description	5074	5104	5231	5278	5284	5330	5333	5334	5335	5337	5341	5342	5343	5344	5345	5431	# Isolates
**Lipoproteins** a																		
spr0884	foldase protein PrsA		×	×	×	×		×		×			×	×				8/16
spr1060	pneumococcal histidine triad protein D precursor	×		×	×	×	×			×		×		×	×			9/16
spr1382	ABC transporter substrate-binding protein - oligopeptide transport (AliB)	×	×	×		×	×			×	×	×		×				9/16
**Cell Wall**																		
spr0057	beta-N-acetylhexosaminidase	×	×	×	×	×	×		×	×	×	×	×	×	×		×	14/16
spr0075	cell wall surface anchor family protein			×	×		×			×	×	×	×		×			8/16
spr0247	alkaline amylopullulanase	×		×	×	×	×	×		×	×		×	×		×		11/16
spr0286	hyaluronate lyase precursor (hyaluronidase/hyase)			×	×	×	×		×	×	×	×	×		×	×	×	12/16
spr0328	cell wall surface anchor family protein	×	×	×		×	×	×	×	×	×	×	×	×	×	×	×	15/16
spr0440	endo-beta-N-acetylglucosaminidase, putative	×	×	×	×	×	×	×		×	×		×	×		×		12/16
spr0561	cell wall-associated serine proteinase precursor PrtA	×	×		×	×	×	×	×	×	×		×	×	×	×		13/16
spr0565	beta-galactosidase precursor	×			×	×		×	×	×		×	×	×	×		×	11/16
spr1403	hypothetical protein spr1403	×	×			×	×	×		×	×	×	×	×	×	×	×	13/16
spr1536	sialidase A precursor (neuraminidase A)	×	×		×	×	×	×	×	×		×	×	×	×	×		13/16
**Membrane proteins (1TMD)**																		
spr0121	surface protein pspA precursor	×	×	×	×	×	×		×	×	×	×	×	×	×		×	14/16
spr0329	penicillin-binding protein 1A			×		×	×	×		×		×	×	×				8/16
spr0334	hypothetical protein spr0334		×	×	×	×				×	×			×			×	8/16
spr0907	pneumococcal histidine triad protein D precursor	×		×	×	×		×	×	×	×	×	×	×	×	×	×	14/16
spr1370	hypothetical protein spr1370		×	×	×	×		×		×	×	×		×			×	10/16
spr1875	hypothetical protein spr1875	×		×		×	×	×		×				×	×			8/16
spr2045	serine protease	×		×	×	×	×	×	×		×		×	×			×	11/16
**Membrane proteins (>1TMD)**																		
SP_0071	immunoglobulin A1 protease	×	×	×	×	×	×					×	×	×				9/16
spr0012	cell division protein FtsH			×	×	×	×	×		×	×	×		×	×	×	×	12/16
spr0086	hypothetical protein spr0086	×	×	×	×	×	×	×		×	×	×			×		×	12/16
spr0581	Zinc metalloprotease	×	×	×	×	×	×	×	×	×	×	×	×	×	×	×	×	16/16
spr1042	immunoglobulin A1 protease	×	×	×	×	×	×	×	×	×	×	×	×	×	×	×	×	16/16
SPN23F_10590	Zinc metalloprotease ZmpD		×			×	×	×	×					×	×		×	8/16
SP670_2141	TMP repeat family		×	×	×	×	×				×			×		×		8/16
**Secretory**																		
spr0908	pneumococcal histidine triad protein E precursor	×	×		×	×		×		×	×		×	×	×			10/16
spr0931	hypothetical protein spr0931		×	×		×	×	×		×	×	×	×	×	×			11/16
spr1903	UTP-glucose-1-phosphate uridylyltransferase	×	×	×	×	×	×			×	×			×		×		10/16
spr1995	choline binding protein A	×	×	×	×	×	×	×	×	×	×	×	×	×	×	×	×	16/16
spr2021	general stress protein GSP-781	×	×		×	×	×	×	×	×	×	×		×	×			12/16

a Protein categories were established according to LocateP subcellular predictions: lipoproteins were those predicted as lipid-anchored proteins; cell wall proteins, as those having an LPXTG motif; secretory proteins, as those possessing an SP1-type signal peptide; membrane proteins with one transmembrane domain (TMD), as those possessing either a *C*- or an *N*-terminally anchored transmembrane region; membrane proteins with more than one transmembrane domain, those predicted as multi-transmembrane proteins; “surface proteins” means the sum of the five previous categories; and cytoplasmic proteins, those without any exporting or sorting signal, and predicted as intracellular proteins.

**Figure 2:**
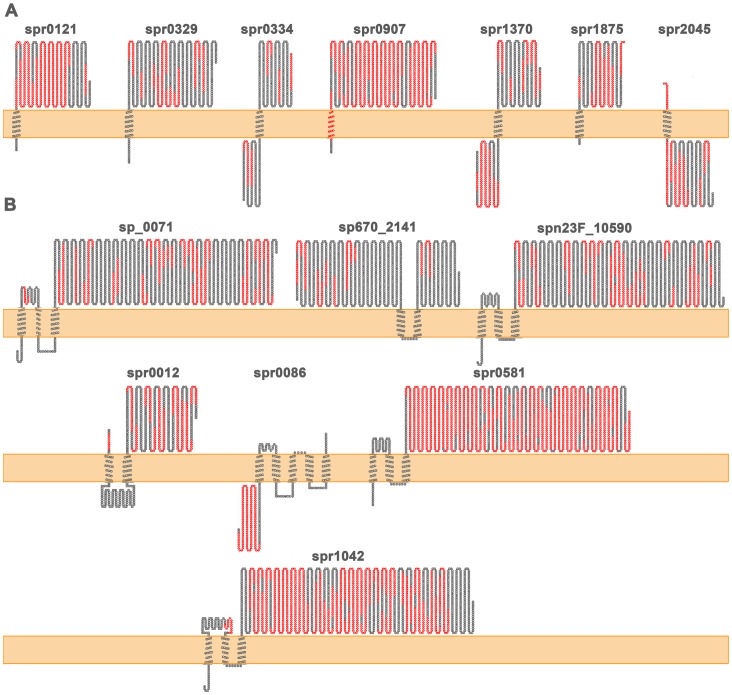
Topology representation of the predicted membrane proteins in the “pan-surfome” of the 16 *S. pneumoniae* clinical isolates that have been identified in at least 50% of the analyzed strains. The TMHMM algorithm was used to predict transmembrane domains (TMD) and signal peptides after prediction of subcellular localization by LocateP. In red are shown the peptides experimentally identified by LC/MS/MS. A) Proteins with only one predicted TMD. B) Proteins with more than one TMD.

Although predicted membrane proteins (i.e. those with ≥1 transmembrane domain) are normally the most abundant surface protein group identified in “shaving” experiments, they represent the lowest percentage compared to the corresponding total number of predicted proteins in databases, compared to the other groups [15], [16], [69]. Cell-wall anchored, secreted proteins and lipoproteins are completely surface-exposed, except some small regions embedded within the cell wall and/or the capsule. Therefore, any portion of them would be *a priori* good for cloning in order to produce recombinant fragments for vaccine testing (in the case they were too large to select the whole sequence). However, membrane proteins are theoretically more embedded under the surface, and therefore their extracellular domains are less exposed. In addition, those with more than one transmembrane domain are generally dificult to obtain in a recombinant way, because of solubility problems. The best strategy to use them in protection assays would consist of selecting those domains that are experimentally confirmed to be surface-accessible. **Figure 2** shows the peptides identified after LC/MS/MS analysis over the topology predictions for the corresponding sequences of the 14 membrane proteins shown in **Table 3**. For 9 proteins, experimental assignments of identified peptides were in agreement with the predicted topologies (i.e. peptides corresponded to extracellular domains). However, for the other 5 proteins (Spr0086, Spr0334, Spr0907, Spr1370 and Spr2045), discrepancies were observed, as we identified peptides belonging to predicted cytoplasmic domains. These disagreements have also been reported in other works [16], [69], [70], although in *S. pyogenes* it has been experimentally demonstrated by flow cytometry assays that some of these predicted cytoplasmic domains are actually surface-located [16], thus showing that prediction algorithms do not always correctly predict the topology of all the membrane proteins. Moreover, a deeper view on the identified peptides for each individual isolate may provide “hot zones” (i.e. those identified in many strains) that might be best for selection of cloning fragments, in case of very large proteins (Dataset S4).

**Figure 3:**
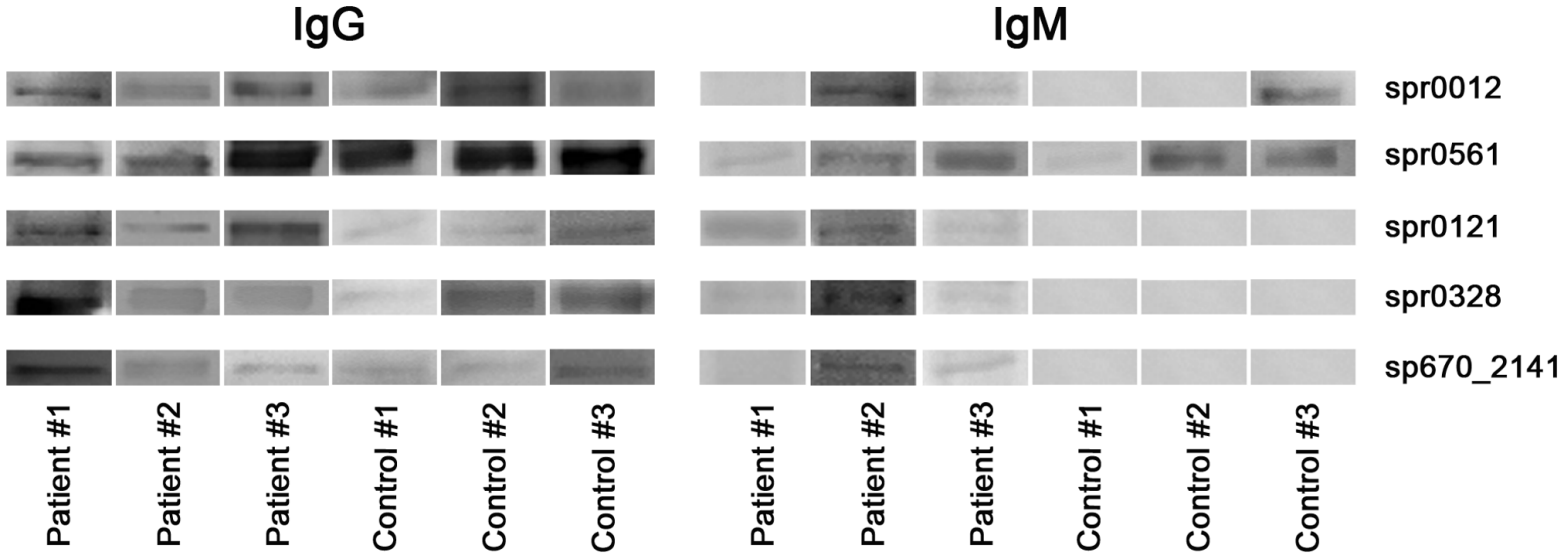
Western blot analysis of five selected surface proteins against human sera. Detection of both IgG (left) and IgM (right) was carried out. In all the cases, antibody reaction was observed against the positive control (pneumococcal protein extract; see Materials and Methods for further details), and no reaction against the negative control (trypsin; see Materials and Methods for further details).

Finally, we evaluated the power of “pan-surfomics” for the selection of protein antigens with vaccine potential, by measuring the immunoreactivity in sera from infected patients. Thus, we selected five proteins from those identified in common in at least 50% of the isolates, and performed a Western-blot analysis to detect both IgG and IgM reaction. Of these proteins, four had not previously been assayed for protection. We also selected PspA (Spr0121), whose protection capacity against infection is well known [53], to validate the utility of this approach. As a positive control, we used a total protein extract from pneumococcus, and as a negative control, commercially available trypsin. As shown in **Figure 3**, IgG antibodies were raised against the five proteins, even in the three sera used as healthy controls, which might be due to previous asymptomatic pneumococcal colonization. Regarding IgM response, two out of the three patients raised these antibodies against the five selected proteins; the other patient (patient #1) produced IgM antibodies against three proteins (Spr0121, Spr0328 and Spr0561). The three healthy donors raised IgM antibodies against Spr0561. In addition, control #3 showed anti-Spr0012 IgM antibodies. As observed in the figure, both the IgG and IgM responses against Spr0561 were very intense, which could mean that it is strongly immunogenic. This high immunogenicity, which may be due to its highly surface exposure and size (it is a 2,144-amino acid cell-wall protein) could be also responsible for the presence of IgM antibodies in healthy donors if they had been previously colonized by pneumococcus.

We have previously shown the utility of the detection of immunoreactivity against selected surface proteins for the discovery of diagnostic biomarkers of infection both in children and in adults [19]. In that study, we demonstrated that five known protective antigens raised antibodies in convalescent patients. In addition, a cell-wall protein, the alkaline pullulanase PulA, not previously tested at protection level, was shown to induce antibody response. Here we show the potential of four novel surface proteins (Spr0012, Spr0328, Spr0561 and SP670_2141) for vaccine testing, because of their capacity to raise immune response in infected patients. As expected, an IgM response against most of, or all the selected proteins, was obtained in the infected patient sera (2 out of 3), but much less in the control individuals (as stated above, only against Spr0561 in the three healthy donors and against Spr0012 in control #3). On the contrary, the five proteins induced IgG production both in the infected and the control people. The detection of IgG antibodies in healthy, control individuals may be explained by non-symptomatic carriage, which can mask a true discrimination among markers of disease. Therefore, IgM detection should be *a priori* a better indicator of antigen exposure. However, the lower levels of these antibodies make them more difficult to detect, and factors such as timing in taking the samples may represent some limitations [71]. More sensitive, large scale formats for IgM-based protein antigen detection are needed, as customized protein chips or Luminex assays.

In conclusion, this study shows the utility of pan-surfomic approach in the high-trhoughput screening of proteins that could enter the pipeline for new vaccine discovery. The selection of the candidates from the proteomic analysis must be complemented with immunogenicity studies to determine the capacity of such proteins to raise an effective immune response. Further research is needed to test the protection capacity of the proposed proteins in animal models of infection, either individually or as “antigen cocktail” formulations. Although the main application field of such an approach is the discovery of new protein vaccines, the development of serological tools for detecting diagnostic biomarkers, and/or the application in programs of epidemiological surveillance, is also very promising, especially if large-scale formats (protein arrays, Luminex assays) are set up.

## Supporting Information

Table S1 Surface proteins identified in the 16 Streptococcus pneumoniae clinical isolates analyzed. Shown are the locus numbers, gi accession numbers, protein function descriptions, subcellular localization according to LocateP, presence of the proteins in each isolate, and number of isolates in which each protein has been found.
(XLSX)

Dataset S1 Recombinant fragments produced in this work from the selected gene products. In gray are highlighted the sequences amplified (from the corresponding genes) and produced as recombinant polypeptides (from the corresponding amino acid sequences). Number of nucleotides amplified and amino acids expressed are indicated for each protein, as well as primers used for DNA amplification.
(PDF)

Dataset S2 Sequest raw data of protein and peptide identifications for *S. pneumoniae* clinical isolates 5074-5, 5104-8, 5231-9V, 5278-14, 5284-9V, 5330-7F, 5333-19A and 5334-8. Each isolate’s data are given separately in one or more datasheets.
(XLS)

Dataset S3 Sequest raw data of protein and peptide identifications for *S. pneumoniae* clinical isolates 5335-5, 5337-7F, 5341-1, 5342-3, 5343-14, 5344-19A, 5345-1 and 5431-12F. Each isolate’s data are given separately in one or more datasheets.
(XLS)

Dataset S4 Representation of the sequences identified belonging to membrane proteins over the 50% threshold (see Table 3), and their frequency in the “pan- surfome” of the 16 *Streptococcus pneumoniae* clinical isolates analyzed.
(PDF)
