# Supplementary material for: Identification of Potential New Protein Vaccine Candidates through Pan-Surfomic Analysis of Pneumococcal Clinical Isolates from Adults
Source: PLoS One. 2013 Jul 23;8(7):e70365. doi: 10.1371/journal.pone.0070365 (PMC3720901; doi:10.1371/journal.pone.0070365)
Supplement: Dataset S1 — Recombinant fragments produced in this work from the selected gene products. In gray are highlighted the sequences amplified (from the corresponding genes) and produced as recombinant polypeptides (from the corresponding amino acid sequences). Number of nucleotides amplified and amino acids expressed are indicated for each protein, as well as primers used for DNA amplification. (PDF) [file pone.0070365.s002.pdf]

Dataset S1: Recombinant fragments produced in this work from the selected gene products. In gray are highlighted the sequences amplified (from the corresponding genes) and produced (from the corresponding proteins).

spr0012. Cell division protein FtsH (EC:3.4.24.-)

aa sequence: 652

MKKQNNGLIKNPFLWLLFIFFLVTGFQYFYSGNNSGGSQQINYTELVQEITDGNVKELTY
QPNGSVIEVSGVYKNPKTSKEGTGIQFFTPSVTKVEKFTSTILPADTTVSELQKLATDHK
AEVTVKHESSSGIWINLLVSIVPFGILFFFLFSMMGNMGGGNGRNPMSFGRSKAKAANKE
DIKVRFSDVAGAEEEKQELVEVVEFLKDPKRFTKLGARIPAGVLLEGPPGTGKTLLAKAV
AGEAGVPFFSISGSDFVEMFVGVGASRVRSLFEDAKKAAPAIIFIDEIDAVGRQRGVGLG
GGNDEREQTLNQLLIEMDGFEGNEGIIVIAATNRSDVLDPALLRPGRFDRKVLVGRPDVK
GREAILKVHAKNKPLAEDVDLKLVAQQTPGFVGADLENVLNEAALVAARRNKSIIDASDI
DEAEDRVIAGPSKKDKTVSQKERELVAYHEAGHTIVGLVLSNARVVHKVTIVPRGRAGGY
MIALPKEDQMLLSKEDMKEQLAGLMGGRVAEEIIFNVQTTGASNDFEQATQMARAMVTEY
GMSEKLGPVQYEGNHAMLGAQSPQKSISEQTAYEIDEEVRSLLNEARNKAAEIIQSNRET
HKLIAEALLKYETLDSTQIKALYETGKMPEAVEEESHALSYDEVKSKMNDEK

Nt sequence: 1959

atgaaaaaacaaaataatggtttaattaaaaatcctttttctatggttattatttatcttt
ttccttgtgacaggattccagtatttctattctgggaataactcaggaggaagtcagcaa
atcaactatactgagttggtacaagaaattaccgatggtaatgtaaaagaattaacttac
caaccaaatggtagtgttatcgaagtttctggtgtctataaaaatcctaaaacaagtaaa
gaaggaacaggtattcagtttttcacgccatctgttactaaggtagagaaatttaccagc
actattcttcctgcagatactaccgtatcagaattgcaaaaacttgctactgaccataaa
gcagaagtaactgttaagcatgaaagttcaagtggtatatggattaatctactcgtatcc
attgtgccatttggaattctattcttcttcctattctctatgatgggaaatatgggagga
ggcaatggccgtaatccaatgagttttggacgtagtaaggctaaagcagcaaataaagaa
gatattaaagtaagattttcagatgttgctggagctgaggaagaaaaacaagaactagtt
gaagttgttgagttcttaaaagatccaaaacgattcacaaaacttggagcccgtattcca
gcaggtgttcttttggagggacctccggggacaggtaagactttgcttgctaaggcagtc
gctggagaagcaggtgttccattctttagtatctcaggttctgactttgtagaaatgttt
gtcggagttggagctagtcgtgttcgctctcttttttgaggatgccaaaaaagcagcacca
gctatcatctttatcgatgaaattgatgctgttggacgtcaacgtggagtcggtctcggc
ggaggtaatgacgaacgtgaacaaaccttgaaccaacttttgattgagatggatggtttt
gagggaaatgaagggattatcgtcatcgctgcgacaaaccgttcagatgtacttgatcct
gcccttttgcgtccaggacgttttgatagaaaagtattggttggccgtcctgatgttaaa
ggtcgtgaagcaatcttgaaagttcacgctaagaacaagcctttagcagaagatgttgat
ttgaaattagtggctcaacaaactccaggctttgttggtgctgatttagagaatgtcttg
aatgaagcagctttagttgctgctcgtcgcaataaatcgataattgatgcttcagatatt
gatgaagcagaagatagagttattgctggaccttctaagaaagataagacagtttcacaa
aaagaacgagaattggttgcttaccatgaggcaggacataccattgttggtctagtcttg
tcgaatgctcgcgttgtccataaggttacaattgtaccacgcggccgtgcaggcggatac
atgattgcacttcctaaagaggatcaaatgcttctatctaaagaagatatgaaagagcaa

ttggctggcttaatgggtggacgtgtagctgaagaaattatctttaatgtccaaactaca
ggagcttcaaacgactttgaacaagcgacacaaatggcacgtgcaatggttacagagtac
ggtatgagtgaaaaacttggcccagtacaatatgaaggaaaccatgctatgcttggtgca
cagagtcctcaaaaatcaatttcagaacaaacagcttatgaaattgatgaagaggttcgt
tcattattaaatgaggcacgaaataaagctgctgaaattattcagtcaaatcgtgaaact
cacaagttaattgcagaagcattattgaaatacgaaacattggatagtacacaaattaaa
gctctttacgaaacaggaaagatgcctgaagcagtagaagaggaatctcatgcactatcc
tatgatgaagtaaagtcaaaaatgaatgacgaaaaataa

Protein	Forward (5'->3')	Reverse (5'->3')
spr0012	TAAT GAT ATC GAA GTT GTT GAG TTC TTA AAA GA	GCTGTA GCG GCC GCA TTT TTC GTC ATT CAT TTT TGA CT

1359 nt cloned

spr0561. Cell wall-associated serine proteinase precursor PrtA (EC:3.4.21.-)

aa sequence: 2144
MKKSTVLSLTTAAVILAAYAPNEVVLADTSSSEDALSISDKEKVVVDKETENKEKHKDIH
NAIETSKDTEEKKTTIIEEKEVVSKNPVIDTKTSNEEAKIKEENSNQSQGDHTDSFVNKN
TENPKKEDKVVYIAEFKDKESGEKAIKGLSNLKNTKVLYTYDRIFNGSAIETTPDNLDKI
KQIEGISSIERAQKVQPMMNHARKEIGVEEAIDYLKSINAPFGKNFDGRGMVISNIDTGT
DYRHKAMRIDDDAKASMRFKKEDLKGTDKNYWLSDKIPHAFNYYNGGKITVEKYDDGRDY
FDPHGMHIAGILAGNDTEQDIKNFNGIDGIAPNAQIFSYKMYSDAGSGFAGDETMFHAIE
DSIKHNVDVVSVSSGFTGTGLVGEKYWQAIRALRKAGIPMVVATGNYATSASSSSWDLVA
NNHLKMTDTGNVTRTAAHEDAIAVASAKNQTVEFDKVNIGGESFKYRNIGAFFDKNKITT
NEDGTKAPSKLKFVYIGKGQDQDLIGLDLRGKIAVMDRIYTKDLKNAFKKAMDKGARAIM
VVNTVNYYNRDNWTELPAMGYEADEGTKSQVFSISGDDGVKLWNMINPDKKTEVKRNNKE
DFKDKLEQYYPIDMESFNSNKPNVGDEKEIDFKFAPDTDKELYKEDIIVPAGSTSWGPRI
DLLLKPDVSAPGKNIKSTLNVINGKSTYGYMSGTSMATPIVAASTVLIRPKLKEMLERPV
LKNLKGDDKIDLTSLTKIALQNTARPMMDATSWKEKSQYFASPRQQGAGLINVANALRNE
VVATFKNTDSKGLVNSYGSISLKEIKGDKKYFTIKLHNTSNRPLTFKVSASAITTDSLTD
RLKLDETYKDEKSPDGKQIVPEIHPEKVKGANITFEHGTFTIGANSSFDLNAVINVGEAK
NKNKFVESFIHFESVEEMEALNSNGKKINFQPSLSMPLMGFAGNWNHEPILDKWAWEEGS
RSKTLGGYDDDGKPKIPGTLNKGIGGEHGIDKFNPAGVIQNRKDKNTTSLDQNPELFAFN
NQGINAPSSSGSKIANIYPLDSNGNPQDAQLERGLTPSPLVLRSAEEGLISIVNTNKEGE
NQRDLKVISREHFIRGILNSKSNDAKGIKSSKLKVWGDLKWDGLIYNPRGREENAPESKD
NQDPATKIRGQFEPIAEGQYFYKFKYRLTKDYPWQVSYIPVKIDNTAPKIVSVDFSNPEK
IKLITKDTYHKVKDQYKNETLFARDQKEHPEKFDEIANEVWYAGAALVNEDGEVEKNLEV
TYAGEGQGRNRKLDKDGNTIYEIKGAGDLRGKIIEVIALDGSSNFTKIHRIKFADQADEK
GMISYYLVDPDKDASKYEKLGEISEDKLKNAKSPEENTNNNQAKDEDSKPDEKSSVEGEA
SLEINKTISTIREFENKDLKKLIKKKFREVNDFTSETGKRIEEYDYKYDDKGNIIAYDDG
SALQYETEKFDEIKSKIYGVLSPSKDGHFEILGKISNVSKNAKVYYGNSYKSIEIKATKY
DSHSKTMIFDLYANINDIVDGLAFAGDMRLFVKDDNQIKAETKIRMPEKNKETKAEYPYV
SSYGNVIELGEGDLSKNKPDNLTKMESGKIYSDSEKQQYLLKDNIILRKGYALKVTTYNP
GKTDMLEGNGVYSKEDIAKIQKANPNLRVLSETTIYADSRNVEDGRSTQAVLMSALDGFN
IIRYQVFTFKMNDKGEAIDKDGNLVTDSSKLVLFGKDDKEYTGEDKSNVEAIKEDGSMLF
IDTKPVNLSMDKNYFNPSKSNKIYVRNPEFYLRGKISDKGGFNWELRVNESVVDNYLIYG
DLHIDNTRDFNIKLNVKDGDIMDWGMKDYKANGFPDKVTDMDGNVYLQTGYSDLNAKAVG
VHYQFLYDNVKPEVNIDPKGNTSIEYADGKSVVFNINDKRNNGFDGEIQEQHIYVNGKEY
TSFDDIKQITDKTLNIKIVVKDFARNTTVKEFILNKDTGEVSELKPHRVTVTIQNGKEMS
STIVSEEDFILPVYKGELEKGYQFDGWEISGFEGKKDAGYVINLSKDTFIKPVFKKIEEK

KEEENKPTFDVSKKKDNPQVNHSQLNESHRKEDLQREDHSQKSDSTKDVTATVLDKNNIS
SKSTTNNPNKLPKTGTASGAQTLLAAGIMFIVGIFLGLKKKNQD

Nt sequence: 6435
atgaaaaaaagcacagtattgtcactaactacagctgcagttattttagcagcctatgcc
cctaatgaggtagtcttagcagacacatctagctctgaagatgctttaagcatctctgat
aaagaaaaagtagtagtagataaggaaacagaaaataaagagaaacataaagatattcat
aatgctatagaaacttcaaaggatactgaagaaaagaaaacaacaattattgaggaaaaa
gaagttgttagtaaaaatcctgtaatagacactaaaactagcaatgaagaagcaaaaatc
aaagaagaaaattccaatcaatcccaaggagatcatacggactcatttgtgaataaaaac
acagaaaatcccaaaaaagaagataaagttgtctatattgctgaatttaaagataaagaa
tctggagaaaaagcaatcaagggactatcaaatcttaagaatacaaaagttttatatact
tatgatagaatttttaacggtagtgccatagaaacaactccagataacttggacaaaatt
aaacaaatagaaggtatttcatcgattgaaagggcacaaaaagtccaacccatgatgaat
catgccagaaaggaaattggagttgaggaagctattgattacctaaagtctatcaatgct
ccgtttgggaaaaattttgatggtagaggtatggtcatttcaaatatcgatactggaaca
gattatagacataaggctatgagaatcgatgatgatgccaaagcctcaatgagatttaaa
aaagaagacttaaaaggaactgataaaaattattggttgagtgataaaatccctcatgcg
ttcaattattataatggtggcaaaatcactgtagaaaaatatgatgatggaagggattat
tttgacccacatgggatgcatattgcagggattcttgctggaaatgatactgaacaagat
atcaaaaactttaacggcatagatggaattgcacctaatgcacaaattttctcttacaaa
atgtattctgacgcaggatctgggtttgcgggtgatgaaacaatgtttcatgctattgaa
gattctatcaaacacaacgttgatgttgtttcggtatcatctggttttacaggaacaggt
cttgtaggtgagaaatattggcaagctattcgggcattaagaaaagcaggcattccaatg
gttgtcgctacgggtaactatgcgacttctgcttcaagttcttcatgggatttagtagca
aataatcatctgaaaatgaccgacactggaaatgtaacacgaactgcagcacatgaggat
gcgatagcggtcgcttctgctaaaaatcaaacagttgagtttgataaagttaacataggt
ggagaaagttttaaatacagaaatatagggcctttttcgataagaataaaatcacaaca
aatgaagatggaacaaaagctcctagtaaattaaaatttgtatatataggcaaggggcaa
gaccaagatttgataggtttggatcttaggggcaaaattgcagtaatggatagaatttat
acaaaggatttaaaaaatgcttttaaaaaagctatggataagggtgcacgcgccattatg
gttgtaaatactgtaaattactacaatagagataattggacagagcttccagctatggga
tatgaagcggatgaaggtactaaaagtcaagtgttttcaatttcaggagatgatggtgta
aagctatggaacatgattaatcctgataaaaaaactgaagtcaaaagaaataataaagaa
gattttaaagataaattggagcaatactatccaattgatatggaaagttttaattccaac
aaaccgaatgtaggtgacgaaaaagagattgactttaagtttgcacctgacacagacaaa
gaactctataaagaagatatcatcgttccagcaggatccacatcttgggggccaagaata
gatttacttttaaaacccgatgtttcagcacctggtaaaaatattaaatctacgcttaat
gttattaatggcaaatcaacttatggctatatgtcaggaactagtatggcgactccaatc
gtggcagcttctactgttttgattagaccgaaattaaaggaaatgcttgaaagacctgta
ttgaaaaatcttaagggagatgacaaaatagatcttacaagtcttacaaaaattgcccta
caaaatactgctcgacctatgatggatgcaacttcttggaaagaaaaaagtcaatacttt
gcatcacctagacaacagggagcaggcctaattaatgtggccaatgctttgagaaatgaa
gttgtagcaactttcaaaaacactgattctaaaggtttggtaaactcatatggttccatt
tctcttaaagaaataaaaggtgataaaaaatactttacaatcaagcttcacaatacatca
aacagacctttgacttttaaagtttcagcatcagcgataactacagattctctaactgac
agattaaaacttgatgaaacatataaagatgaaaaatctccagatggtaagcaaattgtt
ccagaaattcacccagaaaaagtcaaaggagcaaatatcacatttgagcatggtactttc
actataggcgcaaattctagctttgatttgaatgcggttataaatgttggagaggccaaa
aacaaaaataaatttgtagaatcatttattcattttgagtcagtggaagaaatggaagct
ctaaactccaacgggaagaaaataaacttccaaccttctttgtcgatgcctctaatggga
tttgctgggaattggaaccacgaaccaatccttgataaatgggcttgggaagaagggtca
agatcaaaaacactgggaggttatgatgatgatggtaaaccgaaaattccaggaacctta
aataaggaattggtggagaacatggtatagataaatttaatccagcaggagttatacaa
aatagaaaagataaaaatacaacatccctggatcaaaatccagaattatttgctttcaat
aaccaagggatcaacgctccatcatcaagtggttctaagattgctaacatttatccttta

gattcaaatggaaatcctcaagatgctcaacttgaaagaggattaacaccttctccactt
gtattaagaagtgcagaagaaggattgatttcaatagtaaatacaaataaagagggagaa
aatcaaagagacttaaaagtcatttcgagagaacactttattagaggaattttaaattct
aaaagcaatgatgcaaagggaatcaaatcatctaaactaaaagtttggggtgacttgaag
tgggatggactcatctataatcctagaggtagagaagaaaatgcaccagaaagtaaggat
aatcaagatcctgctactaagataagaggtcaatttgaaccgattgcggaaggtcaatat
ttctataaatttaaatatagattaactaaagattacccatggcaggtttcctatattcct
gtaaaaattgataacaccgcccctaagattgtttcggttgatttttcaaatcctgaaaaa
attaagttgattacaaaggatacttatcataaggtaaaagatcagtataagaatgaaacg
ctatttgcgagagatcaaaaagaacatcctgaaaaatttgacgagattgcgaacgaagtt
tggtatgctggcgccgctcttgttaatgaagatggagaggttgaaaaaaatcttgaagta
acttacgcaggtgagggtcaaggaagaaatagaaaacttgacaaagacggaaataccatt
tatgaaattaaaggtgcaggagatttaagaggaaaaatcattgaagtcattgcattagat
ggttctagcaatttcacaaagattcatagaattaaatttgctgatcaggctgatgaaaag
gggatgatttcttattacctagtagatcctgacaaagatgcttctaaatatgaaaaactt
ggtgaaatttctgaagataaactcaaaaatgcaaagagcccagaggaaaataccaataat
aatcaagctaaggatgaagattcaaaaccagatgaaaaaagttcagttgagggagaagct
agccttgaaataaataaaactatttcaacaattagagagtttgaaaataaagacctaaag
aaactcattaaaaagaaatttagagaagttaatgattttacaagtgaaactggtaagaga
atagaggaatacgattataaatacgatgataagggaaatattattgcttatgacgatggt
agtgccttacaatatgaaactgaaaaatttgacgaaataaaatcaaaaatttatggtgtt
ctaagcccatctaaagatggacactttgaaattcttggaaagataagcaatgtttctaaa
aatgccaaggtatattatggaaatagctataaatcgatagaaatcaaagcgactaagtat
gattcacattcaaaaacgatgatatttgatttatacgctaatattaatgatattgtagat
ggattagcttttgctggagatatgagattatttgttaaagatgacaatcaaataaaagct
gaaactaagattagaatgcctgaaaaaaataaggaaactaaagcagaatatccctatgta
tcaagttatgggaatgtaatcgaattaggagaaggagatctttcaaagaacaaaccagac
aatttaactaaaatggaatctggtaaaatctattctgattcagaaaaacaacaatatctg
ttaaaggataacatcattctaagaaaaggctatgcactaaaagtgactacctataatcct
ggaaaaacggatatgttagaaggaaatggagtctatagcaaggaagatatagcaaaaata
caaaaggccaatcctaatctaagagtcctttcagaaacaacaatttatgctgatagtaga
aatgttgaagatggaagaagtacccaagctgtattaatgtcggctttggacggctttaac
attataaggtatcaagtgtttacatttaaaatgaacgataaaggggaagctatcgataaa
gacggaaatcttgtgacagattcttctaaacttgtattatttggtaaggatgataaagaa
tacactggagaggataagtccaatgtagaagctataaaagaagatggctccatgttattt
attgataccaaaccagtaaacctttcaatggataagaactactttaatccatctaaatct
aataaaatttatgtacgaaatccagaattttatttaagaggtaagatttctgataagggt
ggttttaactgggaattgagagttaatgaatcggttgtagataattatttaatctacgga
gatttacacattgataacactagagattttaatattaagctgaatgttaaagacggtgac
atcatggactggggaatgaaagactataaagcaaacggatttccagataaggtaacagat
atggatggaaatgtttatcttcaaactggctatagcgatttgaatgctaaagcagttgga
gtccactatcagtttttatatgataatgttaaacctgaagtaaacattgatcctaaggga
aatactagtatcgaatatgctgatggaaaatctgtagtctttaacatcaatgataaaaga
aataatggattcgatggtgagattcaagaacaacatatttatgtaaatggaaaagaatat
acatcatttgatgatattaaacaaataacagacaagacactaaacattaagattgttgta
aaagattttgcaagaaatacaaccgtaaaagaattcattttaaataaagatacgggagag
gtaagtgaattaaaacctcatagggtaactgtgaccattcaaaatggaaaagaaatgagt
tcaacgatagtgtcggaagaagattttattttacctgtttataagggtgaattagaaaaa
ggataccaatttgatggttgggaaatttctggtttcgaaggtaaaaaagacgctggctat
gttattaatctatcaaaagatacctttataaaacctgtattcaagaaaatagaggagaaa
aaggaggaagagaataaacctacttttgatgtatcgaaaaagaaagataacccacaagta
aatcatagtcaattaaatgaaagtcacagaaaagaggatttacaaagagaagatcattca
caaaaatctgattcaactaaggatgttacagctacagttcttgataaaaacaatatcagt
agtaaatcaactactaacaatcctaataagttgccaaaaactggaacagcaagcggagcc
cagacactattagctgccggaataatgtttatagtaggaatttttcttggattgaagaaa
aaaaatcaagattaa

Protein	Forward (5'->3')	Reverse (5'->3')
spr0561	ATAT TCG CGA GAC ACA TCT AGC TCT GAA GAT	GATGAT GCG GCC GCG AAT AGC TTG CCA ATA TTT CTC

1090 nt cloned

spr0121. surface protein pspA precursor

aa sequence: 619

MNKKKMILTSLASVAILGAGFVASQPTVVRAEESPVASQSKAEKDYDAAKKDAKNAKKAV
EDAQKALDDAKAAQKKYDEDQKKTEEKAALEKAASEEMDKAVAAVQQAYLAYQQATDKAA
KDAADKMIDEAKKREEEAKTKFNTVRAMVVPEPEQLAETKKKSEEAKQKAPELTKKLEEA
KAKLEEAEKKATEAKQKVDAEEVAPQAKIAELENQVHRLEQELKEIDESESEDYAKEGFR
APLQSKLDAKKAKLSKLEELSDKIDELDAEIAKLEDQLKAAEENNNVEDYFKEGLEKTIA
AKKAELEKTEADLKKAVNEPEKPAPAPETPAPEAPAEQPKPAPAPQPAPAPKPEKPAEQP
KPEKTDDQQAEEDYARRSEEEYNRLTQQQPPKAEKPAPAPKTGWKQENGMWYFYNTDGSM
ATGWLQNNGSWYYLNSNGAMATGWLQYNGSWYYLNANGAMATGWAKVNGSWYYLNANGAM
ATGWLQYNGSWYYLNANGAMATGWAKVNGSWYYLNANGAMATGWLQYNGSWYYLNANGAM
ATGWAKVNGSWYYLNANGAMATGWVKDGDTWYYLEASGAMKASQWFKVSDKWYYVNGLGA
LAVNTTVDGYKVNANGEWV

Nt sequence: 1860
atgaataagaaaaaaatgattttaacaagtctagccagcgtcgctatcttaggggctggt
tttgttgcgtctcagcctactgttgtaagagcagaagaatctcccgtagccagtcagtct
aaagctgagaaagactatgatgcagcgaagaaagatgctaagaatgcgaaaaaagcagta
gaagatgctcaaaaggctttagatgatgcaaaagctgctcagaaaaaatatgacgaggat
cagaagaaaactgaggagaaagccgcgctagaaaaagcagcgtctgaagagatggataag
gcagtggcagcagttcaacaagcgtatctagcctatcaacaagctacagacaaagccgca
aaagacgcagcagataagatgatagatgaagctaagaaacgcgaagaagaggcaaaaact
aaatttaatactgttcgagcaatggtagttcctgagccagagcagttggctgagactaag
aaaaaatcagaagaagctaaacaaaaagcaccagaacttactaaaaaactagaagaagct
aaagcaaaattagaagaggctgagaaaaaagctactgaagccaaacaaaaagtggatgct
gaagaagtcgctcctcaagctaaaatcgctgaattggaaaatcaagttcatagactagaa
caagagctcaaagagattgatgagtctgaatcagaagattatgctaaagaaggtttccgt
gctcctcttcaatctaaattggatgccaaaaaagctaaactatcaaaacttgaagagtta
agtgataagattgatgagttagacgctgaaattgcaaaacttgaagatcaacttaaagct
gctgaagaaaacaataatgtagaagactactttaaagaaggtttagagaaaactattgct
gctaaaaaagctgaattagaaaaaactgaagctgaccttaagaaagcagttaatgagcca
gaaaaaccagctccagctccagaaactccagccccagaagcaccagctgaacaaccaaaa
ccagcgccggctcctcaaccagctcccgcaccaaaaccagagaagccagctgaacaacca
aaaccagaaaaaacagatgatcaacaagctgaagaagactatgctcgtagatcagaagaa
gaatataatcgcttgactcaacagcaaccgccaaaagctgaaaaaccagctcctgcacca
aaaacaggctggaaacaagaaaacggtatgtggtacttctacaatactgatggttcaatg
gcgacaggatggctccaaaacaacggttcatggtactacctcaacagcaatggtgctatg
gctacaggttggctccaatacaatggttcatggtattacctcaacgctaacggtgctatg
gcaacaggttgggctaaagtcaacggttcatggtactacctcaacgctaatggtgctatg
gctacaggttggctccaatacaacggttcatggtattacctcaacgctaacggcgctatg
gcaacaggttgggctaaagtcaacggttcatggtactacctcaacgctaatggtgctatg
gctacaggttggctccaatacaacggttcatggtactacctcaacgctaacggtgctatg
gctacaggttgggctaaagtcaacggttcatggtactacctcaacgctaatggtgctatg
gcaacaggttgggtgaaagatggagatacctggtactatcttgaagcatcaggtgctatg
aaagcaagccaatggttcaaagtatcagataaatggtactatgtcaatggtttaggtgcc
cttgcagtcaacacaactgtagatggctataaagtcaatgccaatggtgaatgggtttaa

Protein	Forward (5'->3')	Reverse (5'->3')
spr0121	TAGA GAT ATC GTG GCA GCA GTT CAA CAA GCG	GATACA GCG GCC GCA AAC CCA TTC ACC ATT CGC ATT G

1557 nt cloned

spr0328. Cell wall surface anchor family protein

aa sequence: 1767
MNKGLFEKRCKYSIRKFSLGVASVMIGATFFGTSPVLADSVQSGSTANLPADLATALATA
KENDGHDFEAPKVGEDQGSPEVTDGPKTEEELLALEKEKPAEEKPKEDKPAAAKPETPKT
VTPEWQTVEKKEQQGTVTIREEKGVRYNQLSSTAQNDNAGKPALFEKKGLTVDANGNATV
DLTFKDDSEKGKSRFGVFLKFKDTKNNVFVGYDKDGWFWEYKSPTTSTWYRGSRVAAPET
GSTNRLSITLKSDGQLNASNNDVNLFDTVTLPAAVNDHLKNEKKILLKAGSYDDERTVVS
VKTDNQEGVKTEDTPAEKETGPEVDDSKVTYDTIQSKVLKAVIDQAFPRVKEYSLNGHTL
PGQVQQFNQVFINNHRITPEVTYKKINETTAEYLMKLRDDAHLINAEMTVRLQVVDNQLH
FDVTKIVNHNQVTPGQKIDDERKLLSSISFLGNALVSVSSDQTGAKFDGATMSNNTHVSG
DDHIDVTNPMKDLAKGYMYGFVSTDKLAAGVWSNSQNSYGGGSNDWTRLTAYKETVGNAN
YVGIHSSEWQWEKAYKGIVFPEYTKELPSAKVVITEDANADKKVDWQDGAIAYRSIMNNP
QGWKKVKDITAYRIAMNFGSQAQNPFLMTLDGIKKINLHTDGLGQGVLLKGYGSEGHDSG
HLNYADIGKRIGGVEDFKTLIEKAKKYGAHLGIHVNASETYPESKYFNEKILRKNPDGSY
SYGWNWLDQGINIDAAYDLAHGRLARWEDLKKKLGDGLDFIYVDVWGNGQSGDNGAWATH
VLAKEINKQGWRFAIEWGHGGEYDSTFHHWAADLTYGGYTNKGINSAITRFIRNHQKDAW
VGDYRSYGGAANYPLLGGYSMKDFEGWQGRSDYNGYVTNLFAHDVMTKYFQHFTVSKWEN
GTPVTMTDNGSTYKWTPEMRVELVDADNNKVVVTRKSNDVNSPQYRERTVTLNGRVIQDG
SAYLTPWNWDANGKKLSTDKEKMYYFNTQAGATTWTLPSDWAKSKVYLYKLTDQGKTEEQ
ELTVKDGKITLDLLANQPYVLYRSKQTNPEMSWSEGMHIYDQGFNSGTLKHWTISGDASK
AEIVKSQGANDMLRIQGNKEKVSLTQKLTGLKPNTKYAVYVGVDNRSNAKASITVNTGEK
EVTTYTNKSLALNYVKAYAHNTRRNNATVDDTSYFQNMYAFFTTGSDVSNVTLTLSREAG
DEATYFDEIRTFENNSSMYGDKHDTGKGTFKQDFENVAQGIFPFVVGGVEGVEDNRTHLS
EKHDPYTQRGWNGKKVDDVIEGNWSLKTNGLVSRRNLVYQTIPQNFRFEAGKTYRVTFEY
EAGSDNTYAFVVGKGEFQSGRRGTQASNLEMHELPNTWTDSKKAKKATFLVTGAETGDTW
VGIYSTGNASNTRGDSGGNANFRGYNDFMMDNLQIEEITLTGKMLTENALKNYLPTVAMT
NYTKESMDALKEAVFNLSQADDDISVEEARAEIAKIEALKNALVQKKTALVADDFASLTA
PAQAQEGLANAFDGNLSSLWHTSWGGGDVGKPATMVLKEATEITGLRYVPRGSGSNGNLR
DVKLVVTDESGKEHTFTATDWPDNNKPKDIDFGKTIKAKKIVLTGTKTYGDGGDKYQSAA
ELIFTRPQVAETPLDLSGYEAALAKAQKLTDKDNQEEVASVQASMKYATDNHLLTERMVE
YFADYLNQLKDSATKPDAPTVEKPEFKLSSVASDQGKTPDYKQEIARPETPEQILPATGE
SQFDTALFLASVSLALSALFVVKTKKD

Nt sequence: 5304
atgaataaaggattatttgaaaaacgttgtaaatatagtattcggaaattttcattaggt
gttgcttctgttatgattggagctacattctttgggacaagtccggttcttgcagatagc
gtgcagtctggttccacggcgaacttaccagctgatttagctactgctcttgcaacagca
aaagagaatgatgggcatgattttgaagcgcctaaggtgggagaagaccaaggttctcca
gaagttacagatggacctaagacagaagaagaactattagcacttgaaaaagaaaaaccg
gctgaagaaaaaccaaaagaggataaacctgcagctgctaaacctgaaacacctaagacg
gtaacccctgaatggcaaacggtagagaaaaaagaacaacagggaacagtcactatccga
gaagaaaaaggtgtccgctacaaccaattatcctcaactgctcaaaatgataacgcaggt
aaaccagccctgtttgaaaagaagggcttgaccgttgatgccaatggaaatgcaactgtt
gatttaaccttcaaagatgattctgaaaagggcaaatcacgctttggtgtcttcttgaaa
tttaaagataccaagaataatgtttttgtcggttacgacaaggatggctggttctgggag
tataaatctccaacaactagcacttggtatagaggtagtcgtgttgctgctcctgaaaca
ggatcaacaaaccgtctctctatcactctcaagtcagacggtcagctaaatgccagcaat

aacgatgtcaatctctttgacacagtgactctaccagctgcggtcaatgaccatcttaaa
aatgagaagaagattcttctcaaggcgggctcttatgacgatgagcgaacagttgttagc
gttaaaacggataaccaagaggggtaaaaacagaggataccccctgctgaaaaagaaaca
ggtcctgaagttgatgatagcaaggtgacttatgacacgattcagtctaaggttctcaaa
gcagtgattgaccaagccttccctcgtgtcaaggaatacagcttgaatggacatactttg
ccaggacaggttcaacagttcaaccaagtctttatcaataaccaccgaatcacccctgaa
gtcacttataagaaaatcaatgagacaacagcagagtacttgatgaagcttcgcgatgat
gctcacttaatcaatgcggaaatgacagtacgcttgcaagttgtggacaatcaattgcac
tttgatgtgaccaagattgtcaaccacaatcaagtcactccaggtcaaaagattgatgac
gaaagaaaactactttcttctattagtttcctcggcaatgctttagtctctgtttctagt
gatcaaactggtgctaagtttgatggggcaaccatgtcaaacaatacgcatgtcagcgga
gatgatcatatcgatgtaaccaatccaatgaaagatctagccaagggttacatgtatgga
tttgtttctacagataagcttgctgctggtgtttggagtaactctcaaaacagctatggt
ggtggttcgaatgactggactcgtttgacagcctataaagaaacagtcggaaatgccaac
tatgtaggaatccacagctctgaatggcaatgggaaaaagcttataagggcattgttttc
ccagaatacacgaaggaacttccaagtgctaaggttgttatcactgaagatgccaatgca
gacaagaaagtcgattggcaggatggtgccattgcttatcgtagcattatgaacaatcct
caaggttggaaaaaagttaaggatatcacagcttaccgtatcgcgatgaactttggttct
caagcacaaaacccattccttatgaccttggatggtatcaagaaaatcaatctccacaca
gatggtcttgggcaaggtgttctccttaaaggatatggtagcgaaggccatgactctggt
cacttgaactatgctgatattggtaagcgtatcggtggtgtcgaagacttcaagacccta
attgagaaggctaagaaatatggagctcatctaggtatccacgttaacgcttcagaaact
tatcctgagtctaaatacttcaatgaaaaaattctccgtaagaatccagatggaagctat
agctatggttggaactggctagatcaaggtatcaacattgatgctgcctatgacctagct
catggtcgtttggcacgttgggaagatttgaagaaaaaacttggtgacggtctcgacttt
atctatgtggacgtttggggtaatggtcaatcaggtgataacggtgcctgggctacccac
gttcttgctaaagaaattaacaaacaaggctggcgctttgcgatcgagtggggccatggt
ggtgagtacgactctaccttccatcactgggcagctgacttgacctacggtggctacacc
aataaaggtatcaacagtgccatcacccgctttatacgtaaccaccaaaaagatgcttgg
gtaggggactacagaagttatggtggtgcagccaactatccactgctaggtggctacagc
atgaaagactttgaaggctggcaaggaagaagtgactacaatggctatgtaactaactta
tttgcccatgacgtcatgaccaagtacttccaacacttcactgtaagtaaatgggaaaat
ggtacaccggtgactatgaccgataacggtagcacctataaatggactccagaaatgcga
gtggaattggtagatgctgacaataataaagtagttgtaactcgtaagtcaaatgatgtc
aatagtccacaatatcgcgaacgtacagtaactctcaacggacgtgtcatccaagatggt
tcagcttacttgactccttggaactgggatgcaaatggtaagaaactttctactgataag
gaaaagatgtactacttcaatacgcaggccggtgcaacaacttggacccttccaagcgat
tgggcaaagagcaaggtttacctttacaagctaactgaccaaggtaagacagaagagcaa
gaactaactgtaaaagatggtaaaattaccctagatcttctagcaaatcaaccatacgtt
ctctatcgttcgaaacaaaccaatcctgaaatgtcatggagtgaaggcatgcacatctat
gaccaaggatttaacagtggtaccttgaaacattggaccatttcaggcgatgcttctaag
gcagaaattgtcaagtctcaaggggcaaacgatatgcttcgtattcaaggaaacaaagaa
aaagttagtctcactcagaaattaactggcttgaaaccaaataccaagtatgccgtttat
gtcggtgtcgataaccgtagtaatgccaaggcgagcatcactgtaaatactggtgaaaaa
gaagtgactacttataccaataagtctctcgccctcaactatgtaaaagcctatgcccac
aatacacgtcgtaacaatgctacagttgacgatacaagttacttccaaaacatgtacgcc
ttctttacaactggatcggacgtatcaaatgttactctgacattgagtcgtgaagctggt
gatgaagcaacttactttgatgaaattcgtacctttgaaaacaattcaagcatgtacgga
gacaagcatgatacaggtaaaggcaccttcaagcaagactttgaaaatgttgctcagggt
atcttcccatttgtagtgggtggtgtcgaaggtgtcgaagacaaccgcactcacttgtct
gaaaaacacgatccatatacacaacgtggttggaatggtaagaaagtcgatgatgttatc
gaaggaaattggtcactcaagacaaatggactagtgagccgtcgtaacttggtttaccaa
actattccgcaaaacttccgttttgaagcaggtaagacctaccgtgtaacctttgaatac
gaagcaggttcagacaatacctatgcttttgtagtcggtaagggagaattccagtcaggt
cgtcgtggtactcaagcaagcaacttggaaatgcatgaattgccaaatacttggacagat
tctaagaaagccaagaaggcaaccttcctcgtgacaggtgcagaaacaggggatacttgg

gtaggtatctactcaactggaaatgcaagtaatactcgtggtgattctggtggaaatgcc
aacttccgtggttataacgacttcatgatggataatcttcaaatcgaagaaattaccta
acaggtaagatgttgacagaaaatgctctgaagaactacttgccaacggttgccatgact
aactacaccaaagagtctatggatgctttgaaagaggcggtctttaacctcagtcaggcc
gatgatgatatcagtgtggaagaagcgcgtgcagagattgccaagattgaagccttgaag
aatgctttggttcagaagaaaacggctttggtagcagatgactttgcaagtcttacagct
cctgctcaggctcaagaaggtcttgcaaatgcctttgatggaaacttatctagtttatgg
catacatcatggggcggaggagatgtaggcaagcctgcaaccatggtcttgaaagaagca
actgaaatcactggacttcgttatgttccacgtggatcaggttcaaatggtaacttgcgt
gatgtgaaacttgttgtgacagatgagtctggcaaggagcatacctttactgcaactgat
tggccagataacaataagccaaaagacattgattttggtaagacaattaaggctaagaaa
attgtccttacaggtactaagacttacggagatggtggcgataaataccaatctgcagcg
gaactcatctttactcgtccacaggtagcagaaacacctcttgacttgtcaggctatgaa
gcagctttggctaaggctcagaaattaacagacaaagacaatcaagaggaagtagctagc
gttcaggcaagcatgaaatatgcgacggataaccatctcttgacggaaagaatggtggaa
tactttgcagattatctcaaccaattaaaagattctgctacgaaaccagatgctccaact
gtagagaaacctgagtttaaacttagctctgtagcttccgatcaaggtaagacgccagat
tataagcaagaaatagctagaccagaaacacctgaacaaatcttgccagcaacaggtgag
agtcaatttgacacagccctcttcctagcaagtgttagcctagccctatctgctctcttt
gtagtaaaaacgaagaaagactag

Protein	Forward (5'->3')	Reverse (5'->3')
spr0328	TTTA GAT ATC AGC GTG CAG<br>TCT GGT TCC A	GATGAT GCG GCC GCT ATT GAT<br>AAA GAC TTG GTT GAA C

1003 nt cloned

sp670_2141. TMP repeat family

aa sequence: 1217
MATLDELKVMIDAEIAPFRKKMKEVENQVKGTSDQVKNATAKVREQSSSIGSAFGKLAKF
AGFAILGKKLLDVGMYSTQTALEVSASMNQIKRQMGESSQSFLKWVNDNANAMNMGVGEA
TNYGAVYSNLFSGFIKDTNKLSAYTAKMLQTSAVVAEGSGRTITDVMERIRSGLLGNTEA
IEDLGINVNVAMIESTEAFKKFANGQSWQQLDYQTQQQIRLMAILEQATAKYGDTLSNSV
NGRISLFKSLMKDAALNLGNSMLPIINAIMPVLNSFAMVLKNVTAKLAEFIALMFNKKAT
VKDGVGGAVGDMGNAMKDAAGGAGDLADAVDDAGDSAGGLADNLGDSAKNAKKAAKELLG
LLGFDEINILQKPKDDDAGGSGGGGKGGKGKGGGGGPFKDILPEVELTDMDNKFKSIFDG
LGDKLKGLFDLFKKGFDAAFRPEGIKRIKTALDQIAKTMGEIATDPRVVNAFNRMAEKIA
YALGQVTGSITTIGLGIGVFLAESIANGLGRQKERIIRALVALFDNVGNLSEAVGNIAQD
FSSAFYDVITSTGAVRIGSAIVSTLLSLTSTIVEVGSKLAGSLFKGFEKVVVTSAPKISS
VFQSLLDTVAPVFESIERSVNKFGDGLSRVYDEHVVPAINSIANAFNGLIDIIQILWENS
WQPFAEFLSGVFGVSIEGISDLLGGGLLATLGLLADAIKLVADGFTVFSDWCKENKEPIL
ALITTWQTINFLSWAEQAGGLAGAFSLLGSKVSLIVGGIKNLGLAIKALTFDKLVSFGET
IYLNTLYAKDFVVNSGKTIAQLGKTALELGKSALAWTAHAAKMGLATAAEFAHSVAAGVA
TAATWAFNAALAVLTSPITWIIAAIAALIAIGVLLYQNWDTVVEFAKTAWQGLCDFISGI
CRAIGEFFSGLWTKLQEIFEPIGQWFGEKFQQAWDAIVNIFSGIGEWFSGVFQGAWDAIV
NIFTPIGSWFGQRWADVTSALANIGAWFTDIFQKAWTGLTNIFSKLGLWFGERWADVTSV
LANVSSWFGNMFTSAYNAVKNAFSSIGGFFSGVWSTVQSIFVNAGQKVGSAVGGAFKSAV
NAVLGTIENVVNGFIGMINGVLGVVRNLPGLGWVGSVSTVSLPRLARGGIVDSPTIAMIG
EAGKEAVVPLENTGFIQTLGRVVSSAVVNAMAGISPQGGFSSDGDIVIQIAGHEFGRVAI
QEINKEHERAGQTLLKI

Nt sequence: 3654

Atggcaactcttgatgaattgaaagtcatgattgacgctgagatagcgcctttcaggaag
aagatgaaagaagtcgagaatcaggtcaaaggaacatctgaccaagtgaaaaatgctact
gccaaagttcgtgaacagtcgagctcaatcggtagtgcgtttggcaagctggctaagttc
gctggttttgcaatccttggtaagaaattacttgatgttgggatgtattcaacgcagacg
gctcttgaagtatcagcgtctatgaaccaaatcaagcgacagatgggcgagagttcgcaa
tctttcttaaaatgggttaacgataatgccaacgctatgaatatgggtgtgggtgaggct
accaactacggtgcagtctactcaaacttattttctggatttatcaaagataccaacaag
ctaagcgcctataccgctaagatgttgcagacatcggcagttgttgctgaaggttcaggg
cgcacgattacagacgttatggagcggattcgctcaggtttactagggaacaccgaagcg
attgaggacctaggaatcaacgtcaacgtggctatgattgagtccactgaagcctttaag
aagttcgcaaacggacagagctggcaacagttggattaccaaacccagcaacaaatccgc
cttatggctattctggaacaggctacagccaagtatggggataccttgtctaattctgta
aatggtcgtatcagcctatttaagtcgctgatgaaggacgcagcattgaaccttggtaac
tctatgttaccgattatcaatgccattatgcctgtcttgaactcttttgctatggtctta
aagaacgttactgctaaactcgctgagtttatcgctttgatgttcaacaagaaagcaaca
gtgaaagatggtgttggtggagcagttggagacatgggtaacgccatgaaggatgctgca
ggcggagcaggagaccttgctgacgcagtagacgacgctggagattcagcaggaggactt
gctgataatcttggagactcagccaaaaacgctaagaaagctgctaaagagttgctaggt
cttttgggatttgatgagattaacatcttgcaaaaaccaaaagacgacgatgcaggcggt
tctggaggaggtggcaaaggtggtaaaggaaagggaggcggtggcggacctttcaaagac
atcttgccagaagtcgagttgaccgacatggacaacaaattcaagagcatttttgatggt
cttggagataagctcaaagggttgtttgacctcttcaagaaaggttttgatgcagcattt
agaccagaaggtataaaacgcattaagactgccttagaccaaatagctaagacaatggga
gaaatcgccactgacccaagggttgtgaatgcctttaaccgaatggctgagaaaattgct
tatgctttagggcaagtgacaggctcaataaccactatcgggctaggtatcggtgttttc
cttgccgaaagtattgcaaatggccttggaaggcaaaaagaacgcattatcagggcgcta
gtcgctttgtttgataatgttggtaacctttccgaggcagtaggaaacatagctcaggac
ttttctagtgctttctacgacgtcattacctcaactggtgcggttcgtatcggtagcgct
attgtgtcaactctgttgagtttgacatctaccattgttgaagttggtagtaaattagca
ggaagtttgtttaaaggttttgaaaaagtcgttgtgacaagcgctcctaaaatttcatca
gtcttccaaagtttattagatactgttgcgcctgtatttgagagcattgaaaggtctgtt
aacaaatttggcgatggcttaagtcgtgtttatgatgaacatgtagtccctgctattaac
tctattgctaatgcttttaatgggctaattgacattattcagattctctgggagaattcc
tggcaacctttttgctgagtttttatcaggagtattcggtgttagtattgaaggaatttca
gatttattaggaggtggcctttttagccactttgggactattggcggatgctattaagtta
gtggcagatggtttcaccgtttttttctgactggtgtaaagaaaacaaagaacctatcttg
gctttgataacaacttggcaaacgattaatttcttatcatgggctgaacaagccgggga
cttgcaggagcattcagcttgttaggtagtaaggtctctttgattgttggagggattaag
aatctaggtcttgctattaaagcattgacatttgataagttggtcagttttggtgaaaca
atctatttgaacaccttatatgcaaaagattttgtggtcaattcaggtaaaacaattgca
cagctaggaaaaactgctttagaacttggtaaatcagctctagcatggactgctcatgca
gcgaaaatgggattagcaaccgcggcggaatttgcacattctgttgcagcaggagtcgct
acagctgcaacatgggcttttaatgcagcgttagcagttttgacaagtccaataacatgg
attattgcagcaatcgcagctttgattgctatcggtgtcttgctctaccaaaactgggac
actgttgttgagtttgctaaaactgcatggcaaggactatgtgattttattagtggtatt
tgtcgagctattggcgaatttttcagtggtctatggacaaaactacaagaaatctttgag
ccgataggtcaatggtttggcgagaagttccagcaagcatgggatgccattgtcaacatc
ttctctggtatcggagagtggttctctggtgtattccaaggtgcatgggacgctatcgtt
aatatcttcactccaatcggctcatggtttggacaacgttgggcagatgtgactagtgct
ttggctaatatcggggcatggtttactgacatattccaaaaagcatggactggtctaaca
aacatctttagcaaactaggtttatggtttggcgagagatgggcagatgttacaagtgtt
cttgcgaatgtatcttcttggtttgggaatatgtttactagtgcttataatgcagtcaag
aacgcgtttagttcaattggtggcttcttcagcggtgtatggtcaacggttcaaagcata
tttgtcaatgctggacaaaaggttggtagcgctgtaggtggagcgtttaagagtgcggtc
aatgcggttcttggaacgattgaaaatgtagtcaatggcttcatcggaatgattaatgga

gttttaggcgttgtcagaaacttacctggtctaggatgggttggtagtgtaagcacagtt
agcctccctcgtcttgcccgtggtggtatcgtcgatagtccaacaatcgccatgattggt
gaagctggtaaagaggcggtcgtaccacttgaaaatacaggatttatccaaacacttgga
cgagtagtcagcagtgcggtagtaaatgccatggctggtattagtccacaaggtggtttt
tctagcgacggcgacatcgttattcaaatcgcaggccatgagttcggacgggtagctatc
caagaaatcaacaaggaacatgaacgagcaggtcaaaccttgctcaagatttag

Protein	Forward (5'->3')	Reverse (5'->3')
sp670_2141	CCCG GAT ATC GCA ACT CTT GAT GAA TTG AAA G	AAAGCA GCG GCC GCG GTT AAT CTC ATC AAA TCC CAA AA

1100 nt cloned
