# Supplementary material for: Identification of Potential New Protein Vaccine Candidates through Pan-Surfomic Analysis of Pneumococcal Clinical Isolates from Adults
Source: PLoS One. 2013 Jul 23;8(7):e70365. doi: 10.1371/journal.pone.0070365 (PMC3720901; doi:10.1371/journal.pone.0070365)
Supplement: Dataset S4 — Representation of the sequences identified belonging to membrane proteins over the 50% threshold (see Table 3), and their frequency in the “pan- surfome” of the 16 Streptococcus pneumoniae clinical isolates analyzed. (PDF) [file pone.0070365.s005.pdf]

Dataset S2: Representation of the sequences identified belonging to membrane proteins over the 50% threshold (Table 3), and their frequency in the "pan-surfome" of the 16 *Streptococcus pneumoniae* clinical isolates analyzed.

Code:

Sequence identified in # isolates	Colour
1	X...X
2	X...X
3	X...X
4	X...X
5	X...X
6	X...X
7	X...X
8	X...X
9	X...X
10	X...X
11	X...X

GI15902165
spr0121
surface protein pspA precursor [Streptococcus pneumoniae R6]

MNKKKMILTS LASVAILGAG FVTSQPTFVR AEEAPVASQS KAEKDYDAAV
KKSEAAKKHY EEAKKKAKEA QKKYDEDQKK TEDKAEAVKK ADEELQKANL
AVQKAYVEYR EAKDKASAEK KIEEAKRKQK EANKKFNEEQ AKVVPEAKEL
AATKQKAEKA KKDAEVAKEK YDKAVQEVEV EKNKILEQDA ENEKKIDVLQ
NKVADLEKGI ALYQNKVAEL NKEIARLQSD LKDAEENNVE DYIKEGLEQA
IADKKAELAT TQQNIDKTQK DELDAELELE KVLATLDPEG KTQDELDKEA
AEDANIEALQ NKVADLENKV AELDKEVTRL QSDLKDAEEN NVEDYVKEGL
EKALTDKKVE LNNTQKALDT AQKALDTALN ELGPDGDEEE TPAPAPKPEQ
PAEQPKPAPA PQPAPAPKPE KTDDQQAEED YARRSEEEYN RLPQQQPPKA
EKPAPAPKPE QPVPAPKPGW KQENGMWYFY NTDGSMATGW LQNNGSWYYL
NSNGAMATGW LQYNGSWYYL NANGAMATGW AKVNGSWYYL NANGAMATGW
LQYNDSWYYL NANGAMATGW LQYNGSWYYL NANGAMATGW AKVNGSWYYL
NANGAMATGW LQYNGSWYYL NANGAMATGW AKVNGSWYYL NANGSMATGW
VKDGDTWYYL EASGAMKASQ WFKVSDKWYY VNGLGALAVN TTVDGYKVNA
NGEWV

GI15902373
spr0329
penicillin-binding protein 1A [Streptococcus pneumoniae R6]

MNKPTILRLI KYLSISFLSL VIAAIVLGGG VFFYYVSKAP SLSESKLVAT
TSSKIYDNKN QLIADLGSER RVNAQANDIP TDLVKAIVSI EDHRFFDHRG
IDTIRILGAF LRNLQSNSLQ GGSALTQQLI KLTYFSTSTS DQTISRKAQE
AWLAIQLEQK ATKQEILTYY INKVYMSNGN YGMQTAAQNY YGKDLNNLSL

PQLALLAGMP QAPNQYDPYS HPEAAQDRRN LVLSEMKNQG YISAEQYEKA
VNTPITDGLQ SLKSASNYPA YMDNYLKEVI NQVEEETGYN LLTTGMDVYT
NVDQEAQKHL WDIYNTDEYV AYPDDELQVA STIVDVSNGK VIAQLGARHQ
SSNVSFGINQ AVETNRDWGS TMKPITDYAP ALEYGVYEST ATIVHDEPYN
YPGTNTPVYN WDRGYFGNIT LQYALQQSRN VPAVETLNKV GLNRAKTFLN
GLGIDYPSIH YSNAISSNTT ESDKKYGASS EKMAAAYAAF ANGGTYYKPM
YIHKVVFSDG SEKEFSNVGT RAMKETTAYM MTDMMKTVLS YGTGRNAYLA
WLPQAGKTGT SNYTDEEIEN HIKTSQFVAP DELFAGYTRK YSMAVWTGYS
NRLTPLVGNG LTVAAKVYRS MMTYLSEGSN PEDWNIPEGL YRNGEFVFKN
GARSTWSSPA PQQPPSTESS SSSSDSSTSQ SSSTTPSTNN STTTNPNNNT
QQSNTTPDQQ NQNPQPAQP

GI15902378

spr0334

hypothetical protein spr0334 [Streptococcus pneumoniae R6]

MSKKRRNRHK KEAQEPQFDF DEAKELTVGQ AIRKNEEVEA GVLPEDSILD
KYVKQHRDEI EADKFATRQY KKEEFVETQS LDDLIQEMRE AVEKSEASSE
EVPSSEDILL PLPLDDEEQG LDPLLLDDEN PTEMTEEVEE EQNLSRLDQE
DSEKKSKKGF ILTVLALVSV IICVSAYYVY RQVARSTKEI ETSQSTTANQ
SDVDDFNTLY DAFYTNSNKT ALKNSQFDKL SQLKTLLDKL EGSREHTLAK
SKYDSLATQI KAIQDVNAQF EKPAIVDGVL DTNAKAKSDA KFTDIKTGNT
ELDKVLDKAI SLGKSQQTST SSSSSSQTSS SSSSQASSNT TSEPKPSSSN
ETRSSRSEVN MGLSSAGVAV QRSASRVAYN QSAIDDSNNS AWDFADGVLE
QILATSRSRG YITGDQYILE RVNIVNGNGY YNLYKPDGTY LFTLNCKTGY
FVGNGAGHAD DLDY

GI15902951

spr0907

pneumococcal histidine triad protein D precursor [Streptococcus pneumoniae R6]

MKINKKYLAG SVAVLALSVC SYELGRHQAG QVKKESNRVS YIDGDQAGQK
AENLTPDEVS KREGINAEQI VIKITDQGYV TSHGDHYHYY NGKVPYDAII
SEELLMKDPN YQLKDSDIVN EIKGGYVIKV DGKYYVYLKD AAHADNIRTK
EEIKRQKQER SHNHNSRADN AVAAARAQGR YTTDDGYIFN ASDIIEDTGD
AYIVPHGDHY HYIPKSDLSA SELAAAQAYW NGKQGSRPSS SSSHNANPAQ
PRLSENHNLT VTPTYHQNQG ENISSLLREL YAKPLSERHV ESDGLIFDPA
QITSRTANGV AVPHGDHYHF IPYSQLSPLE EKLARIIPLR YRSNHWVPDS
RPEQPSPQST PEPSPSPQPA PNPQPAPSNP IDEKLVKEAV RKVGDGYVFE
ENGVPRYIPA KDLSAETAAG IDSKLAKQES LSHKLGAKKT DLPSSDREFY
NKAYDLLARI HQDLLDNKGR QVDFEALDNL LERLKDVSSD KVKLVDDILA
FLAPIRHPER LGKPNAQITY TDDEIQVAKL AGKYTTEDGY IFDPRDITSD
EGDAYVTPHM THSHWIKKDS LSEAERAAAQ AYAKEKGLTP PSTDHQDSGN
TEAKGAEAIY NRVKAAKKVP LDRMPYNLQY TVEVKNGSLI IPHYDHYHNI
KFEWFDEGLY EAPKGYSLED LLATVKYYVE HPNERPHSDN GFGNASDHVQ
RNKNGQADTN QTEKPNEEKP QTEKPEEDKE HDEVSEPTHP ESDEKENHVG
LNPSADNLYK PSTDTEETEE EAEDTTDEAE IPQVEHSVIN AKIAEAEALL
EKVTDSSIRQ NAVETLTGLK SSLLLGTKDN NTISAEVDSL LALLKESQPT
PIQ

GI15903413

spr1370

hypothetical protein spr1370 [Streptococcus pneumoniae R6]

MSEKSREEEK LSFKEQILRD LEKVKGYDEV LKEDEAVVRT PANEPSAEEL
MADSLSTVEE IMRKAPTVPT HPSQGVPASP ADEIQRETPG VPSHPSQDVP
SSPAEESGSR PGPGPVRPKK LEREYNETPT RVAVSYTTAE KKAEQAGPET
PTPATETVDI IRDTSRRSRR EGAKPAKPKK EKKSHVKAFV ISFLVFLALL
SAGGYFGYQY VLDSLLPIDA NSKKYVTVGI PEGSNVQEIG TTLEKAGLVK
HGLIFSFYAK YKNYTDLKAG YYNLQKSMST EDLLKELQKG GTDEPQEPVL
ATLTIPEGYT LDQIAQTVGQ LQGDFKESLT AEAFLAKVQD ETFISQAVAK
YPTLLESLPV KDSGARYRLE GYLFPATYSI KESTTIESLI DEMLAAMDKN
LSLYYSTIKS KNLTVNELLT IASLVEKEGA KTEDRKLIAG VFYNRLNRDM
PLQSNIAILY AQGKLGQNIS LAEDVAIDTN IDSPYNVYKN VGLMPGPVDS
PSLDAIESSI NQTKSDNLYF VADVTEGKVY YANNQEDHDR NVAEHVNSKL
N

GI15903916
spr1875
hypothetical protein spr1875 [Streptococcus pneumoniae R6]

MKKRMLLAST VALSFAPVLA TQAEEVLWTA RSVEQIQNDL TKTDNKTSYT
VQYGDTLSTI AEALGVDVTV LANLNKITNM DLIFPETVLT TTVNEAEEVT
EVEIQTPQAD SSEEVTTATA DLTTNQVTVD DQTVQVADLS QPIAEAPKEV
ASSSEVTKTV IASEEVAPST GTSVPEEQTA ETSSAVAEEA PQETTPAEKQ
ETQTSPQAAS AVEATTTSSE AKEVASSNGA TAAVSTYQPE ETKIISTTYE
APAAPDYAGL AVAKSENAGL QPQTAAFKEE IANLFGITSF SGYRPGDSGD
HGKGLAIDFM VPERSELGDK IAEYAIQNMA SRGISYIIWK QRFYAPFDSK
YGPANTWNPM PDRGSVTENH YDHVHVSMNG

GI15904086

spr2045

serine protease [Streptococcus pneumoniae R6]

MEANMKHLKT FYKKWFQLLV VIVISFFSGA LGSFSITQLT QKSSVNNSNN
NSTITQTAYK NENSTTQAVN KVKDAVVSVI TYSANRQNSV FGNDDTDTDS
QRISSEGSGV IYKKNDKEAY IVTNNHVING ASKVDIRLSD GTKVPGEIVG
ADTFSDIAVV KISSEKVTTV AEFGDSSKLT VGETAIAIGS PLGSEYANTV
TQGIVSSLNR NVSLKSEDGQ AISTKAIQTD TAINPGNSGG PLINIQGQVI
GITSSKIATN GGTSVEGLGF AIPANDAINI IEQLEKNGKV TRPALGIQMV
NLSNVSTSDI RRLNIPSNVT SGVIVRSVQS NMPANGHLEK YDVITKVDDK
EIASSTDLQS ALYNHSIGDT IKITYYRNGK EETTSIKLNK SSGDLES

GI15900016

SP_0071

immunoglobulin A1 protease [Streptococcus pneumoniae TIGR4]

MSRKSIGEKR HSFSMRKLSV GLVSVTVSSF FLMSQGIQSV SADNMESPIH
YKYMTEGKLT DEEKSLLVEA LPQLAEESDD TYYLVYRSQQ FLPNTGFNPT
VGTFLFTAGL SLLVLLVSKR ENGKKRLVHF LLLTSMGVQL LPASAFGLTS
QILSAYNSQL SIGVGEHLPE PLKIEGYQYI GYIKTKKQDN TELSRTVDGK
YSAQRDSQPN STKTSDVVHS ADLEWNQGQG KVSLQGEASG DDGLSEKSSI
AADNLSSNDS FASQVEQNPD HKGESVVRPT VPEQGNPVSA TTVQSAEEEV
LATTNDRPEY KLPLETKGTQ EPGHEGEAAV REDLPVYTKP LETKGTQGPG
HEGEAAVREE EPAYTEPLAT KGTQEPGHEG KATVREETLE YTEPVATKGT
QEPEHEGEAA VEEELPALEV TTRNRTEIQN IPYTTEEIQD PTLLKNRRKI
ERQGQAGTRT IQYEDYIVNG NVVETKEVSR TEVAPVNEVV KVGTLVKVKP
TVEITNLTKV ENKKSITVSY NLIDTTSAYV SAKTQVFHGD KLVKEVDIEN
PAKEQVISGL DYYTPYTVKT HLTYNLGENN EENTETSTQD FQLEYKKIEI
KDIDSVELYG KENDRYRRYL SLSEAPTDTA KYFVKVKSDR FKEMYLPVKS
ITENTDGTYK VTVAVDQLVE EGTDGYKDDY TFTVAKSKAE QPGVYTSFKQ
LVTAMQSNLS GVYTLASDMT ADEVSLGDKQ TSYLTGAFTG SLIGSDGTKS
YAIYDLKKPL FDTLNGATVR DLDIKTVSAD SKENVAALAK AANSANINNV
AVEGKISGAK SVAGLVASAT NTVIENSSFT GKLIANHQDS NKNDTGGIVG
NITGNSSRVN KVRVDALIST NARNNNQTAG GIVGRLENGA LISNSVATGE
IRNGQGYSRV GGIVGSTWQN GRVNNVVSNV DVGDGYVITG DQYAAADVKN
ASTSVDNRKA DRFATKLSKD QIDAKVADYG ITVTLDDTGQ DLKRNLREVD
YTRLNKAEAE RKVAYSNIEK LMPFYNKDLV VHYGNKVATT DKLYTTELLD
VVPMKDDEVV TDINNKKNSI NKVMLHFKDN TVEYLDVTFK ENFINSQVIE
YNVTGKEYIF TPEAFVSDYT AITNNVLSDL QNVTLNSEAT KKVLGAANDA
ALDNLYLDRQ FEEVKANIAE HLRKVLAMDK SINTTGDGVV EYVSEKIKNN
KEAFMLGLTY MNRWYDINYG KMNTKDLSTY KFDFNGNNET STLDTIVALG
NSGLDNLRAS NTVGLYANKL ASVKGEDSVF DFVEAYRKLF LPNKTNNEWF
KENTKAYIVE MKSDIAEVRE KQESPTADRK YSLGVYDRIS APSWGHKSML
LPLLTLPEES VYISSNMSTL AFGSYERYRD SVDGVILSGD ALRTYVRNRV
DIAAKRHRDH YDIWYNLLDS ASKEKLFRSV IVYDGFNVKD ETGRTYWARL
TDKNIGSIKE FFGPVGKWYE YNSSAGAYAN GSLTHFVLDR LLDAYGTSVY
THEMVHNSDS AIYFEGNGRR EGLGAELYAL GLLQSVDSVN SHILALNTLY
KAEKDDLNRL HTYNPVERFD SDEALQSYMH GSYDVMYTLD AMEAKAILAQ
NNDVKKKWFR KIENYYVRDT RHNKDTHAGN KVRPLTDEEV ANLTSLNSLI
DNDIINRRSY DDSREYKRNG YYTISMFSPV YAALSNSKGA PGDIMFRKIA
YELLAEKGYH KGFLPYVSNQ YGAEAFASGS KTFSSWHGRD VALVTDDLVF
KKVFNGEYSS WADFKKAMFK QRIDKQDNLK PITIQYELGN PNSTKEVTIT
TAAQMQQLIN EAAAKDITNI DRATSHTPAS WVHLLKQKIY NAYLRTTDDF
RNSIYK

GI15902056

spr0012

cell division protein FtsH [Streptococcus pneumoniae R6]

MKKQNNGLIK NPFLWLLFIF FLVTGFQYFY SGNNSGGSQQ INYTELVQEI
TDGNVKELTY QPNGSVIEVS GVYKNPKTSK EGTGIQFFTP SVTKVEKFTS
TILPADTTVS ELQKLATDHK AEVTVKHESS SGIWINLLVS IVPFGILFFF
LFSMMGNMGG GNGRNPMSFG RSKAKAANKE DIKVRFSDVA GAEEEKQELV

EVVEFLKDPK RFTKLGARIP AGVLLEGPPG TGKTLLAKAV AGEAGVPFFS
ISGSDFVEMF VGVGASRVRS LFEDAKKAAP AIIFIDEIDA VGRQRGVGLG
GGNDEREQTL NQLLIEMDGF EGNEGIIVIA ATNRSDVLDP ALLRPGRFDR
KVLVGRPDVK GREAILKVHA KNKPLAEDVD LKLVAQQTPG FVGADLENVL
NEAALVAARR NKSIIDASDI DEAEDRVIAG PSKKDKTVSQ KERELVAYHE
AGHTIVGLVL SNARVVHKVT IVPRGRAGGY MIALPKEDQM LLSKEDMKEQ
LAGLMGGRVA EEIIFNVQTT GASNDFEQAT QMARAMVTEY GMSEKLGPVQ
YEGNHAMLGA QSPQKSISEQ TAYEIDEEVR SLLNEARNKA AEIIQSNRET
HKLIAEALLK YETLDSTQIKALYETG KMPE AVEEESHALS YDEVKSKMND
EK

GI15902130

spr0086

hypothetical protein spr0086 [Streptococcus pneumoniae R6]

MKQEWFESND FVKTTSKNKP EEQAQEVADK AEETIADLDT PIEKNTQLEE
EVSQAEVELE SQQEEKIETP EDSEAR TKIE EKKASNSTEE EPDLSKETEK
VTIAEESQEA LPQQKATTKE PLLISKSLES PYIPDQAPKS RDKWKEQVLD
FWSWLVEAIK SPTSKLETSI THSYTAFLLL ILFSASSFFF SIYHIKHAYY
GHIASINSRF PEQLAPLTLF SIVSILVATT LFFFSFLLGS FVVRRFIHQE
KDWTLDKVLQ QYSQLLAIPI FLTAIASFFA FFDSLRFTAL LCVISIGIIL
LASLHIITRP SQASETDSFY QLFLSVLVNG VIILLFFVAE VALIGDYLRI
LAFL

GI15902625

spr0581

Zinc metalloprotease [Streptococcus pneumoniae R6]

MFKKDRFSIR KIKGVVGSVF LGSLLMAPSV VDAATYHYVN KEIISQEAKD
LIQTGKPDRN EVVYGLVYQK DQLPQTGTEA SVLTAFGLLT VGSLLLIYKR
KKIASVFLVG TMGLVVLPSA GAVDPVATLA LASREGVVEM EGYRYVGYLS
GDILKTLGLD TVLEETSAKP GEVTVVEVET PQSTTNQEQA RTENQVVETE
EAPKEEAPKT EESPKEEPKS EVKPTDDTLP KVEEGKEDSA EPAPVEEVGG
EVESKPEEKV AVKPESQPSD KPAEESKVEQ AGEPVAPRKD EQAPVEPENQ
PEAPEEEKAV EETPKQEEST PDTKAEETVE PKEETKTAKG TQEEGKEGQA
PVQEVNPEYK VTTGTVEKST ESELDFTTEV VPDDTKYVDE EVVERQGSKG
VQVTKTTYET VEVVETDKVL STTTEVKTPV VPKVVKKGTK PVETREEVIP
FATKEQEDDT LKRGTRQVAQ EGVNGKKQIT ETYKTIRGEK TNEAPTVEET
VLQAPQDEII KKGTKGLEKP TLQWANTEKD VLKKSATASY TLTKPAGVEI
KSIKLALKDK DGQLVKEVTV AENNLNATLD KLKYYQGYTL STTMVYDRGE
GEETEKLEDK QIQLDLKKVE IKNIKETSLM NVDAEGNETD KSLLSEKPTD
VSQLYLRVTT HDNKVTRLAV SSVEEVVVDG KTLYKVVAKA PDLVQRRADD
TLSEEYVHYF EKQLPKVNNV YYNFNELVKD MQANPMGEFK LGADLNAVNV
KPAGKAYVMA KFRGTLSSVE NHQYTIHNLE RPLFNEAEGA TLKNFNLGNV
NINMPWADKV APIGNMFKKS TLENIKVVGS VTGNNDVTGA VNKLDEANMR
NVAFIGKINS LGDKGWWSGG LVSESWRSNT DSVYFDGDIV GNNSKFGGLV
AKVNHGSNQW DVKQKGRLTN SVVKGTMTLK NHGQSGGLVH ENYDWGWVEN
NISMMKVNNG EIMYGSGSID GDPYFGFDYF KNNYYVKDVA TGESTYKRSK

```
QIQSISQAEA DAKIANMGIT ANTFAIQDPV VNKLNRIIDR DSEYKAIQDY
QETRNLAYRN LEKLQPFYNK EWIVNQGNKL TDESNLVKKT VLSVTGMKSG
QFVTDLSSVD KIMIHYADGT KEEFGVSAIS DSRVKQVKEY NVDDLGVVYT
PNMVDKNRDS LITKVKEKLS SVALDSAEVK SITNNPASLY LEESFAEVRE
TLDKLVKSLL ENEDHQLNSD EVAEKALLKK VEDNKAKIIL ALTYLNRYYG
IDYDGLNFKH LMMFKPDFYG KTPSILDFLI RIGSAEKNLK GDRSLEAYRE
VIGGTIGKGE LNGLLGYNMR LFTKYTDLND WFIHAAKNVY VSEPETTTED
FKDKRHRIYD GLNNDVHGRM ILPLLNLKKA HIFVISTYNT IAFSSFEKYG
KNTEEERNAY KAEIDRVAKA QQRYLDFWSR LALPKVRNQL LKSQNSVPTP
VWDNQVYVGL GGANRMGYGD GGRVVTPVRE LFGPTDRWHQ INWNMGAMAK
IYERPWKDDQ VYFMVTNMME PFGISAFTHE TTHVNDRMAY YGGDWHREGT
DLEAFAQGML QTPDKSTTNG EYGALGINMA YERKNDGEQL YNYDPEKLDS
REKIDSYMKN YNESMMMLDY LEASAVIRQN LSDNSKWFKK MDKEWRTNAD
RNRLIGEPHQ WDKLRDLTEE EKKLPIDSID KLVENNFVTL HGMPKNGRYR
TEGFDSSYQP VNMMAGVFGG NTSKSTVGSI SFKHNAFRMW GYYGYENGFI
PYVSNKLKGA ANKENKGLLG DDFIIKKVSK NQFQNLEEWK KHWYHEVYDK
AQKGFVEIEV DGVKISTYAQ LQSLFEEAVS KDLAGMDDKN IKNHYQYTEN
LKWKIYKQLL KNTDGFSSDL FTAPQA
```

GI15903086

spr1042

immunoglobulin A1 protease [Streptococcus pneumoniae R6]

```
MEKYFGEKQE RFSFRKLSVG LVSATISSLF FMSVLASSSV DAQETAGVHY
KYVADSELSS EEKKQLVYDI PTYVENDDET YYLVYKLNSQ NQLAELPNTG
SKNERQALVA GASLAALGIL IFAVSKKKVK NKTVLHLVLV AGMGNGVLVS
VHALENHLLL NYNTDYELTS GEKLPLPKEI SGYTYIGYIK EGKTTSDFEV
SNQEKSAATP TKQQKVDYNV TPNFVDHPST VQAIQEQTPV SSTKPTEVQV
VEKPFSTELI NPRKEEKQSS DSQEQLAEHK NLETKKEEKI SPKEKTGVNT
LNPQDEVLSG QLNKPELLYR EETIETKIDF QEEIQENPDL AEGTVRVKQE
GKLGKKVEIV RIFSVNKEEV SREIVSTSTT APSPRIVEKG TKKTQVIKEQ
PETGVEHKDV QSGAIVEPAI QPELPEAVVS DKGEPEVQPT LPEAVVTDKG
ETEVQPESPD TVVSDKGEPE QVAPLPEYKG NIEQVKPETP VEKTKEQGPE
KTEEVPVKPT EETPVNPNEG TTEGTSIQEA ENPVQPAEES TTNSEKVSPD
TSSENTGEVS SNPSDSTTSV GESNKPEHND SKNENSEKTV EEVPVNPNEG
TVEGTSNQET EKPVQPAEET QTNSGKIANE NTGEVSNKPS DSKPPVEESN
QPEKNGTATK PENSGNTTSE NGQTEPEKKL ELRNVSDIEL YSQTNGTYRQ
HVSLDGIPEN TDTYFVKVKS SAFKDVYIPV ASITEEKRNG QSVYKITAKA
EKLQQELENK YVDNFTFYLD KKAKEENTNF TSFSNLVKAI NQNPSGTYHL
AASLNANEVE LGPDERSYIK DTFTGRLIGE KDGKNYAIYN LKKPLFENLS
GATVEKLSLK NVAISGKNDI GSLANEATNG TKIKQVHVDG VLAGERGVGG
LLAKADQSSI AESSFKGRIV NTYETTDAYN IGGLVGHLTG KNASIAKSKA
TVTISSNTNR SDQTVGGLAG LVDQDAHIQN SYAEGDINNV KHFGKVAGVA
GYLWDRTSGE EKHAGELTNV LSDVNVTNGN AITGYHYTGM KVANTFSSKA
NRVFNVTLEK DEVVSKESFE ERGTMLDASQ IVSKKAEINP LTLPTVEPLS
TSGKKDSDFS KIAHYQANRA LVYKNIEKLL PFYNKSTIVK YGNLVKENSL
LYQKELLSAV MMKDDQVITD IVSNKQTANK LLLHYNDHSS EKFDLKYQTD
FANLAEYNLG NTGLLYTPNQ FLYDRDSIVK EVLPELQKLD YQSDAIRKTL
GISPEVKLTE LYLEDQFSKT KQNLGDSLKK LLSADAGLAS DNSVTRGYLV
DKIKNNKEAL LLGLTYLERW YNFNYGQVNV KDLVMYHPDF FGKGNTSPLD
TLIELGKSGF NNLLAKNNVD TYGISLASQH GATDLFSTLE HYRKVFLPNT
SNNDWFKSET KAYIVEEKST IEEVKTKQGL AGTKYSIGVY DRITSATWKY
RNMVLPLLTL PERSVFVIST MSSLGFGAYD RYRSSDHKAG KALNDFVEEN
ARETAKRQRD HYDYWYRILD EQSREKLYRT ILLYDAYKFG DDTTSGKATA
EAKFDSSNPA MKNFFGPVGN KVVHNQHGAY ATGDGVYYMS YRMLDKDGAI
```

TYTHEMTHDS DQDIYLGGYG RRNGLGPEFF AKGLLQAPDQ PSDATITINS
ILKHSKSDST EGSRLQVLDP TERFQNAADL QNYVHNMFDL IYMMEYLEGQ
SIVNKLSVYQ KMAALRKIEN KYVKDPADGN EVYATNVVKE LTEAEARNLN
SFESLIDHNI LSAREYQSGD YERNGYYTIK LFAPIYSALS SEKGTPGDLM
GRRIAYELLA AKGFKDGMVP YISNQYEEDA KQQGQTINLY GKERGLVTDE
LVLKKVFDGK YKTWAEFKTA MYQERVDQFG NLKQVTFKDP TKPWPSYGTK
TINNVDELQA LMDQAVLKDA EGPRWSNYDP EIDSAVHKLK RAIFKAYLDQ
TNDFRSSIFE NKK

GI221231868

SPN23F_10590

Zinc metalloprotease ZmpD [Streptococcus pneumoniae ATCC 700669]

MSLLKKDKFS IRKIKGIVGS VFLGSLLFAP SVVGASTYHY LDYSSLTQTE
RDQLKQGRPD ESKESYALDY EKDALPNTGS SQSIMTALGL LAIGSLIVII
TKDNRNKKIA TFLIVGATGL VTLSTASALN LNANIHESGR DGVLQISGYR
YVGYLELDDK TVSSVSPAST VSPVEQPKVV TEKGEPEVHE KPDYTQPIGA
NLVEPEVHEK LAYTEPVGTT GVDENGNLIE PPVNDIPEYT EPVGTTGVDE
NGNLIEPPVS DIPEYTEPIS TVSEVASERE ELPSLHTDIR TETIPKTTIE
ESDPSKFIGD DSVRQVGEDG ERQIVTSYEE LHGKKISDPV ETVTILKEMK
PKILVKGTKE KPKEKTAPVL TLDRTNTNVL NRSATLSYHL VNTDGVTINK
ITATIKDGNE IVKTVDLTSE QLDKQVEDLK FYKDYKIETT MTYDRGKGEE
TATLEEKPLR LDLKKVEIKN IASTNLVKVN DDGTETPSDF MTEKPSDEDV
KKMYLKITSR DNKVTRLAVD KIEEVTEEGK KLYKITAEAQ DLIQHTDPTK
VRNKYVHYIE KPVPKVDDVY YNFKELVDAM NADKNGTFKI GADLNATNVP
TPNKQYVPGT FKGHLSSVDG KQYTIHNIAR PLFDRVENGS VKNINLGNVD
INMPWADGIA PVANMVKNAT VEDVKVTGNV VANNIAGIV NKIDSGGQLT
NVAFIGNLTG VGDKGQYMAG IAGEIWRGNL AKAYVEADIV ANRARIGGLV
AKTDNGNDSM GIGKYGSIRK SVTKGTIKTK VLFETGGFIN SNLPFGKLED
NISMMRVENG EEFFGSSDLD YDGGYFTNGW LERNFVVKGV SSGKHSYKRS
RDKIKEISQD EANKRIANFG LTADKYEINE PVVNRLNRLT RREDEYKSTQ
DYKSERDLAY RNIEKLQPFY NKEWIVNQGN KLAEDSNLAK KEVLSVTGMK
DGQFVTDLSD IDKIMVHYAD GTKEEMDVTK NTDSKVQQVR EYSVSGLGDV
VYTPNMVVKN RDKLIADVKS QLSSVELISQ EVRDLMSRRD KPAENTDERK
NGYIKDLYLE ESFAEVKQNL DKLVKSLVEN EDHQLNGDEA AIKSLLKKVE
TNKAKIMMAL TYLNRYYDIK YGDISIKNIM MFKPDFYGKT PSVIDRLINI
GSSEKNLKGD RTQDAYREII AGNTGKSNLR NFLEYNMRLF TEDKDINDWF
IHSAKNVYVS EPKTTNTELK DKRHRVFDGL DNGVHGRMIL PLLTLKDAHM
FLISTYNTMA YSSFEKYGKH TEEARNEFKT KIDEVAHAQQ TYLDFWSRLA
LPNVRDRLLK SQNMVPTPVW DNQTYNGSPV GRRGFDSKGN PIAPIRELYG
PTWRHHDRDW RMGAMASIFP NPNNDDKVLF MVTDMISPFG ISAFTHETTH
VNDRMLYFGG HKHRQGTDVE AYAQGMLQTP DSSTTNGEYG ALGINMAYHR
PNDGNQWYNP DPDKLKTRDD IDRYMRNYNE AMMLLDHVEA DAVLPKIKGD
NSKWFKKIDK EMRSK IQYND LLGPNQWDSI RDLKDEEKVM TLSSVNDLVD
NNFMTKHGNP GNGRYRPEDF TPNSAYVNVN MMAGIYGGNT SQGAPGSLSF
KHNAFRMWGY YGYENGFISY VSNKYKAEAD KNNHGLLSDK LIINKVSKGN
FNTLEEWKRH WYGEVLAKAK KGFEAIDIDG VHISNYDELR PLFDKAVEED
LKKPDDFSHT VALKSKVFKA LLKNTDGFFN KLFKEDI

GI307128262

SP670_2141

TMP repeat family [Streptococcus pneumoniae 670-6B]

```
MATLDELKVM IDAEIAPFRK KMKEVENQVK GTSDQVKNAT AKVREQSSSI
GSAFGKLAKF AGFAILGKKL LDVGMYSTQT ALEVSASMNQ IKRQMGESSQ
SFLKWVNDNA NAMNMGVGEA TNYGAVYSNL FSGFIKDTNK LSAYTAKMLQ
TSAVVAEGSG RTITDVMERI RSGLLGNTEA IEDLGINVNV AMIESTEAFK
KFANGQSWQQ LDYQTQQQIR LMAILEQATA KYGDTLSNSV NGRISLFKSL
MKDAALNLGN SMLPIINAIM PVLNSFAMVL KNVTAKLAEF IALMFNKKAT
VKDGVGGAVG DMGNAMKDAA GGAGDLADAV DDAGDSAGGL ADNLGDSAKN
AKKAAKELLG LLGFDEINIL QKPKDDDAGG SGGGGKGGKG KGGGGGPFKD
ILPEVELTDM DNKFKSIFDG LGDKLKGLFD LFKKGFDAAF RPEGIKRIKT
ALDQIAKTMG EIATDPRVVN AFNRMAEKIA YALGQVTGSI TTIGLGIGVF
LAESIANGLG RQKERIIRAL VALFDNVGNL SEAVGNIAQD FSSAFYDVIT
STGAVRIGSA IVSTLLSLTS TIVEVGSKLA GSLFKGFEKV VVTSAPKISS
VFQSLLDTVA PVFESIERSV NKFGDGLSRV YDEHVVPAIN SIANAFNGLI
DIIQILWENS WQPFAEFLSG VFGVSIEGIS DLLGGGLLAT LGLLADAIKL
VADGFTVFSD WCKENKEPIL ALITTWQTIN FLSWAEQAGG LAGAFSLLGS
KVSLIVGGIK NLGLAIKALT FDKLVSFGET IYLNTLYAKD FVVNSGKTIA
QLGKTALELG KSALAWTAHA AKMGLATAAE FAHSVAAGVA TAATWAFNAA
LAVLTSPITW IIAAIAALIA IGVLLYQNWD TVVEFAKTAW QGLCDFISGI
CRAIGEFFSG LWTKLQEIFE PIGQWFGEKF QQAWDAIVNI FSGIGEWFSG
VFQGAWDAIV NIFTPIGSWF GQRWADVTSA LANIGAWFTD IFQKAWTGLT
NIFSKLGLWF GERWADVTSV LANVSSWFGN MFTSAYNAVK NAFSSIGGFF
SGVWSTVQSI FVNAGQKVGS AVGGAFKSAV NAVLGTIENV VNGFIGMING
VLGVVRNLPG LGWVGSVSTV SLPRLARGGI VDSPTIAMIG EAGKEAVVPL
ENTGFIQTLG RVVSSAVVNA MAGISPQGGF SSDGDIVIQI AGHEFGRVAI
QEINKEHERA GQTLLKI
```
